# Mesenchymal stem cell-derived exosomes have altered microRNA profiles and induce osteogenic differentiation depending on the stage of differentiation

**DOI:** 10.1371/journal.pone.0193059

**Published:** 2018-02-15

**Authors:** Xiaoqin Wang, Omar Omar, Forugh Vazirisani, Peter Thomsen, Karin Ekström

**Affiliations:** 1 Department of Biomaterials, Institute of Clinical Sciences, Sahlgrenska Academy, University of Gothenburg, Gothenburg, Sweden; 2 BIOMATCELL, VINN Excellence Center of Biomaterials and Cell Therapy, Gothenburg, Sweden; Augusta University, UNITED STATES

## Abstract

Human mesenchymal stem cell (hMSC)-derived exosomes have shown regenerative effects, but their role in osteogenesis and the underlying mechanism are yet to be determined. In this study, we examined the time-course secretion of exosomes by hMSCs during the entire process of osteogenic differentiation. Exosomes derived from hMSCs in various stages of osteogenic differentiation committed homotypic cells to differentiate towards osteogenic lineage, but only exosomes from late stages of osteogenic differentiation induced extracellular matrix mineralisation. Exosomes from expansion and early and late stages of osteogenic differentiation were internalised by a subpopulation of hMSCs. MicroRNA profiling revealed a set of differentially expressed exosomal microRNAs from the late stage of osteogenic differentiation, which were osteogenesis related. Target prediction demonstrated that these microRNAs enriched pathways involved in regulation of osteogenic differentiation and general mechanisms how exosomes exert their functions, such as “Wnt signalling pathway” and “endocytosis”. Taken together, the results show that MSCs secrete exosomes with different biological properties depending on differentiation stage of their parent cells. The exosomal cargo transferred from MSCs in the late stage of differentiation induces osteogenic differentiation and mineralisation. Moreover, it is suggested that the regulatory effect on osteogenesis by exosomes is at least partly exerted by exosomal microRNA.

## Introduction

Exosomes, with a diameter of 30–150 nm [1], are the only subgroup of extracellular vesicles (EVs) known to be derived from endosomes and released into the extracellular milieu upon the fusion of multivesicular bodies with the plasma membrane [2, 3]. Exosomes are secreted by different cell types and exist in most body fluids. The contents of exosomes include lipids, proteins and nucleic acids, such as mRNAs, microRNAs and long non-coding RNA [1, 4–6]. The constituents are highly dependent on the cell type of origin and the microenvironment. Exosomes have attracted considerable attention in recent years due to their capacity to mediate intercellular communication via the transfer of biologically active components, thereby influencing the phenotype and function of target cells [4]. Moreover, exosomes have been shown to be involved in both physiological and pathological processes [7, 8]. The multiple and comprehensive functions of exosomes have resulted in an accumulated interest in using exosomes as biomarkers for diseases or as vectors for cell-free therapy [9].

Mesenchymal stem cells (MSCs) are of great interest as candidates for cell therapy in tissue repair and regeneration, due to their capacity to differentiate to multiple lineage cells and to modulate inflammation/immune responses. The primary strategy has been to transplant MSCs at the site of injury [10]. However, accumulating evidence indicates that there is a lack of correlation between functional improvement and cell engraftment or differentiation at the injury site, suggesting that MSCs exert their therapeutic effects through paracrine secretion [11]. Exosomes are one of the many factors secreted by MSCs and they have been shown to possess promising potency to contribute to regenerative processes *in vitro* and *in vivo* in various organ systems [12]. The application of MSC-derived exosomes may therefore provide a novel strategy for tissue engineering and regenerative medicine.

MicroRNAs are ~22 nt endogenous small non-coding RNAs that function as negative regulators of post-transcriptional gene expression. After processing by enzymes Drosha and Dicer, microRNAs are recruited into the RNA-induced silencing complex (RISC) [13]. The mature microRNAs are then guided to recognize their target mRNAs through perfectly or imperfectly binding to the complementary sequences present in the 5’ end ‘seed’ region or 3’ untranslated regions of target mRNAs, which lead to the degradation or translation inhibition of target mRNAs [14]. The process of bone regeneration via the osteogenic differentiation of MSCs into mature osteoblasts and the subsequent mineralisation are delicately regulated by various microRNAs [15, 16]. Furthermore, the dysfunction of microRNA and the deregulation of microRNA-mediated mechanisms are emerging as important factors in bone degeneration and bone-related diseases such as osteoporosis [17, 18]. The network formed by microRNAs, transcription factors and cell signalling pathways increases the complexity of regulation mechanisms in bone regeneration, while providing various opportunities for the therapeutic modulation of bone regeneration.

Exosomes contain microRNAs with biological functions [19]. It has been suggested that exosomal microRNAs are an important population of extracellular circulating microRNAs involved in the regulation of both physiological and pathological processes [20, 21]. Exosomes secreted from various sources of MSCs have been shown to enrich microRNAs and may be shuttled to target cells, thereby regulating the function of target cells [19, 22–26]. Previous studies have revealed an MSC-derived, exosome-mediated transfer of endogenous miR-133b to neural cells, which promoted neural plasticity and functional recovery from stroke [19, 23]. In addition, MSC-derived exosomes delivered exogenous miR-124 to neural cells in a cell contact-independent manner, resulting in the differentiation of recipient neural cells [25]. Further, genetically modified MSC-derived exosomes were found to mirror the high expression of a specific microRNA, miR-221, in the parent cells and the transfer of miR-221 via exosomes partially mediated the enhancement of cardioprotection [24]. Taken as whole, previous studies have indicated that MSC-derived exosomal microRNAs may play important roles in the biological functions mediated via exosomes.

In the present study, we aimed to determine whether exosomes derived from MSCs (i) are secreted by MSCs during osteogenic differentiation, (ii) become internalised by target MSCs and influence osteogenic differentiation in a stage-dependent manner and (iii) contain different microRNA profiles related to osteogenic differentiation and exosome function, thereby providing underlying, tentative regulatory mechanisms of action.

## Materials and methods

### hMSCs expansion and osteogenic differentiation

hMSCs (ATCC, Manassas, VA, USA) were cultured in exosome-free medium prepared according to Thery *et al*. [27]. For expansion, hMSCs were seeded at 5,000 cells/cm^2^ in basal growth media (BGM), including exosome-free media with 10 ng/ml of human basic fibroblast growth factor (bFGF, Thermo Fisher Scientific, Waltham, MA, USA). To induce osteogenic differentiation, hMSCs were seeded at 18,000 cells/cm^2^ in osteogenic differentiation media (ODM, including exosome-free media supplemented with 100 nM dexamethasone, 45 μM ascorbic acid and 20 mM β-glycerophosphate) in 2 μg/cm^2^ human fibronectin (hFN, BD Biosciences, Erembodegem, Belgium) coated tissue culture flasks (BD Biosciences) for 21 d. Fresh culture medium was replaced every third day and the conditioned medium (CM) was collected following centrifugation at 500 x g for 10 min to eliminate cells. All cell culture experiments were performed three times and three batches of CM were collected. All the CM were stored at -80°C until exosome isolation.

### Exosome isolation

Exosomes were isolated from the CM by a series of differential centrifugation with filtration. First, CM was centrifuged at 16,500 x g for 20 min and filtrated through a 0.22 μm filter to deplete cell debris and large vesicles. Finally, exosomes were pelleted by ultracentrifugation at 120,000 x g for 70 min in a T-647.5 rotor (Sorvall wx Ultra series, Thermo Scientific, USA). The exosome pellets were resuspended in phosphate buffer saline (PBS) and stored at -80°C until use. All steps were performed at 4°C.

### Transmission electron microscopy (TEM)

Ten microliters of exosomes from expansion (Passage 6; P6), early (day 3; D3) and late (day 21; D21) osteogenic differentiation of hMSCs respectively were loaded onto formvar carbon-coated grids (Ted Pella Inc, Redding, CA, USA), fixed in 2% formaldehyde, washed and immunolabelled with anti-CD63 (BD Biosciences) antibody followed by 10 nm gold-labelled secondary antibody (Sigma Aldrich, St Louis, MO, USA). The exosomes were post-fixed in 2.5% glutaraldehyde, washed, contrasted with 2% uranyl acetate and air-dried before TEM examination (Tecnai F20, FEI Company, The Netherlands).

### Nanoparticle tracking analysis (NTA)

To determine the size distribution and particle concentration, three representative groups of exosome samples (Exo_P6, Exo_D3 and Exo_D21, n = 3) were diluted with PBS and analysed by NTA using the NanoSight LM10/LM14 system (NanoSight Ltd, Malvern, UK). Three 60-second videos were captured for each sample. A syringe pump was used for automatic injection before each capture. The setting of the instrument was kept constant for all experiments and videos were analysed using particle tracking (Nanosight software 3.2). The size distribution profiles and particle concentration were averaged within each group of exosomes across all nine video replicates. The results were expressed as the average concentration per μl ± SEM and the average modal size ± SEM (nm) of the nanoparticles.

### Exosome treatment of hMSCs

P6 hMSCs were seeded at a density of 18,000 cells/cm^2^ in hFN-coated 24-well plates in exosome-free medium without any stimulus. After overnight attachment, the culture medium was replaced by medium containing exosomes (n = 3) from hMSCs expansion and different time points (D3, D6, D9, D12, D15, D18 and D21) of osteogenic differentiation. The exosomes used for treatment were isolated from 180 ml of CM of each group; exosomes were re-suspended in 700 μl of PBS. Exosomes (5 μl/well) were added daily to culture medium and the medium was replaced every third day. The ratio of donor cells and recipient cells was approximately 10:1. hMSCs cultured in exosome-free medium without stimulus served as negative control (NCtrl_D0 and NCtrl_D14/D21), while cells cultured in ODM served as positive control (PCtrl). To determine the effects of serum exosomes remaining in the home-made exosome-free medium, serum exosomes (Sexo_Ctrl) were isolated from 180 ml of fresh exosome-free medium and used for hMSC treatment. Each group of hMSC treatment and downstream functional analysis were performed in duplicate for each of three biological replicates.

### Alkaline phosphatase (ALP) activity analysis

After 14 d of treatment, hMSCs were rinsed with DMEM and lysed using M-PER^®^ Protein Extraction Reagent buffer (Thermo Fisher Scientific). The ALP activity (n = 3) was measured using p-nitrophenylphosphate as the substrate. The quantity of p-nitrophenol produced was considered directly proportional to the ALP activity. The data are expressed in enzyme activity, μKat per litre. The ALP activity analysis was performed at the accredited laboratory at Sahlgrenska University Hospital (Gothenburg, Sweden).

### Extracellular matrix (ECM) mineralisation quantification

Calcium and phosphate levels, indicating ECM mineralisation, were quantified after 21 d of treatment. hMSCs were rinsed with PBS and fixed in Histofix^™^ (HistoLab Products AB, Västra Frölunda, Sweden) for 30 min. The cells were washed with distilled water and demineralised by incubation in 0.6 M HCl on an orbital shaker for 24 h at room temperature. The supernatant (n = 3) was collected to measure the calcium and phosphate levels using the ortho-cresolphthalein complexone method and the colorimetric assay of phospho-vanado-molybdic acid respectively. The quantification of ECM calcium and phosphate was performed at the accredited laboratory at Sahlgrenska University Hospital.

### Alizarin Red staining

To further evaluate the mineralisation of hMSCs after exosome treatment for 21 d, cells were washed and fixed as above. After fixation, the cells were washed with distilled water and incubated with Alizarin Red solution (pH 4.0–4.3) for 5 min. The extra dye was removed by rinsing with distilled water. Samples were observed using light microscopy (Nikon TE2000-U, Tokyo, Japan).

### Labelling of exosomes and confocal microscopy

To examine whether hMSCs internalise exosomes derived from hMSCs undergoing osteogenic differentiation, P6 hMSCs were seeded on chamber slides (Millipore, Massachusetts, USA) at a density of 20,000 cells/cm^2^ and cultured overnight. Exosomes (Exo_P6, Exo_D3 and Exo_D21) were labelled with a PKH67 Green Fluorescent Cell Linker Kit for General Cell Membrane Labelling (Sigma-Aldrich) [28]. PKH67-labelled samples were diluted in culture medium and added to hMSCs in culture. After 24 h, cells were washed with PBS, fixed with 2% formaldehyde for 15 min and washed again before mounting with Vectashield HardSet Mounting Medium with DAPI (Vector Laboratories, Burlingame, USA) and visualised using confocal microscopy (Nikon C2 Confocal, Tokyo, Japan).

### RNA extraction and microRNA profiling

The total RNA of exosomes (Exo_P6, Exo_D3 and Exo_D21) and their parent cells was extracted using a miRCURY^™^ RNA Isolation Kit (Exiqon, Vedbaek, Denmark), according to the manufacturer’s protocol (n = 3). To control the quality of the RNA extraction and cDNA synthesis for the microRNA qPCR experiment, a miRCURY LNA^™^ Universal RT microRNA PCR RNA Spike-in kit (Exiqon) was used. The RNA quality was assessed using an Agilent 2100 Bioanalyzer (Agilent Technologies, Foster City, CA, USA). All RNA samples were then submitted to Exiqon for microRNA profiling using the miRCURY LNA^™^ Universal RT microRNA PCR Human panel I containing 372 microRNAs (Exiqon). The microRNA raw data were background filtered and normalised with the global mean Cq value to correct for potential overall differences between the samples.

### MicroRNA target prediction and pathway analysis

Differentially expressed microRNAs, between exosomes from hMSCs expansion and osteogenic differentiation and exosomes from the early (D3) and late (D21) stages of hMSCs osteogenic differentiation, were further analysed to predict their target genes and pathways. The target genes of candidate miRNAs were predicted based on two algorithms, DIANA-microT-CDS and DIANA-TarBase v 7.0 [29]. The microT threshold was set at a score of 0.8 when microT-CDS algorithms were utilised for target gene prediction.

DIANA-mirPath v.3 was applied to perform the hierarchical clustering of miRNAs and all known KEGG pathways based on their interaction levels using predicted miRNA targets provided by the DIANA-microT-CDS algorithm and/or experimentally validated miRNA interactions derived from DIANA-TarBase v7.0. The option gene union in the software was selected to merge the results. The graphical output of the program provides an overview of the pathways modulated by selected miRNAs, facilitating the interpretation and presentation of the analysis results. The statistical significance value associated with the identified biological pathways was calculated automatically by the mirPath software, in which Benjamini and Hochberg's false discovery rate was applied with the significant threshold set at a p-value of < 0.05. The software is available at http://snf-515788.vm.okeanos.grnet.gr/dianauniverse/index.php?r=mirpath.

### Statistics

The difference in the size and concentration of nanoparticles in exosome samples was analysed using the Kruskal-Wallis Test. The exosome treatment experiment was regarded as a randomised block experiment with groups as treatments and biological batches as blocks. The data were analysed with the Proc Mixed SAS procedure and comparisons between groups were made using the estimate statement (SAS version 9.3, SAS Institute Inc., Cary, N.C., USA). The corrections for mass significance were made using the Bonferroni method. The corrected p-value of < 0.05 was considered statistically significant. The data were presented as the mean ± SEM. The microRNA microarray raw data were background filtered and normalised based on the average of the assays detected in all the samples. The normalised dCq values of the top 50 microRNAs with the highest standard deviation were used for the generation of a heat map showing microRNA profiles in both hMSCs and exosomes. A pairwise t test was performed to compare the microRNA expression between different exosome groups. Differentially expressed microRNAs were sorted using a cut-off p-value of < 0.05.

## Results

### Characterisation of hMSCs-derived exosomes

**Fig 1:**
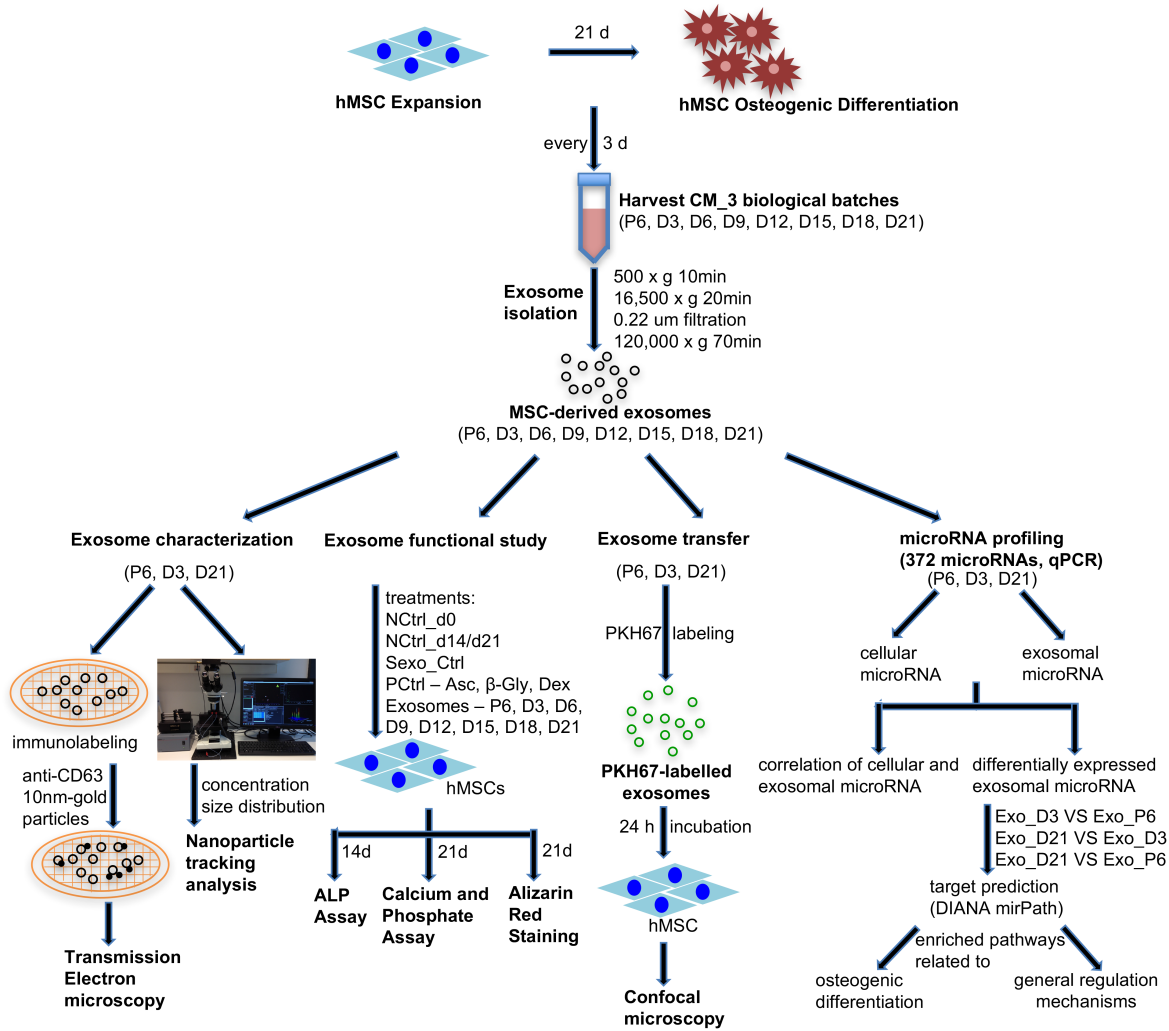
Schematic illustration of the experimental set-up.

**Fig 2:**
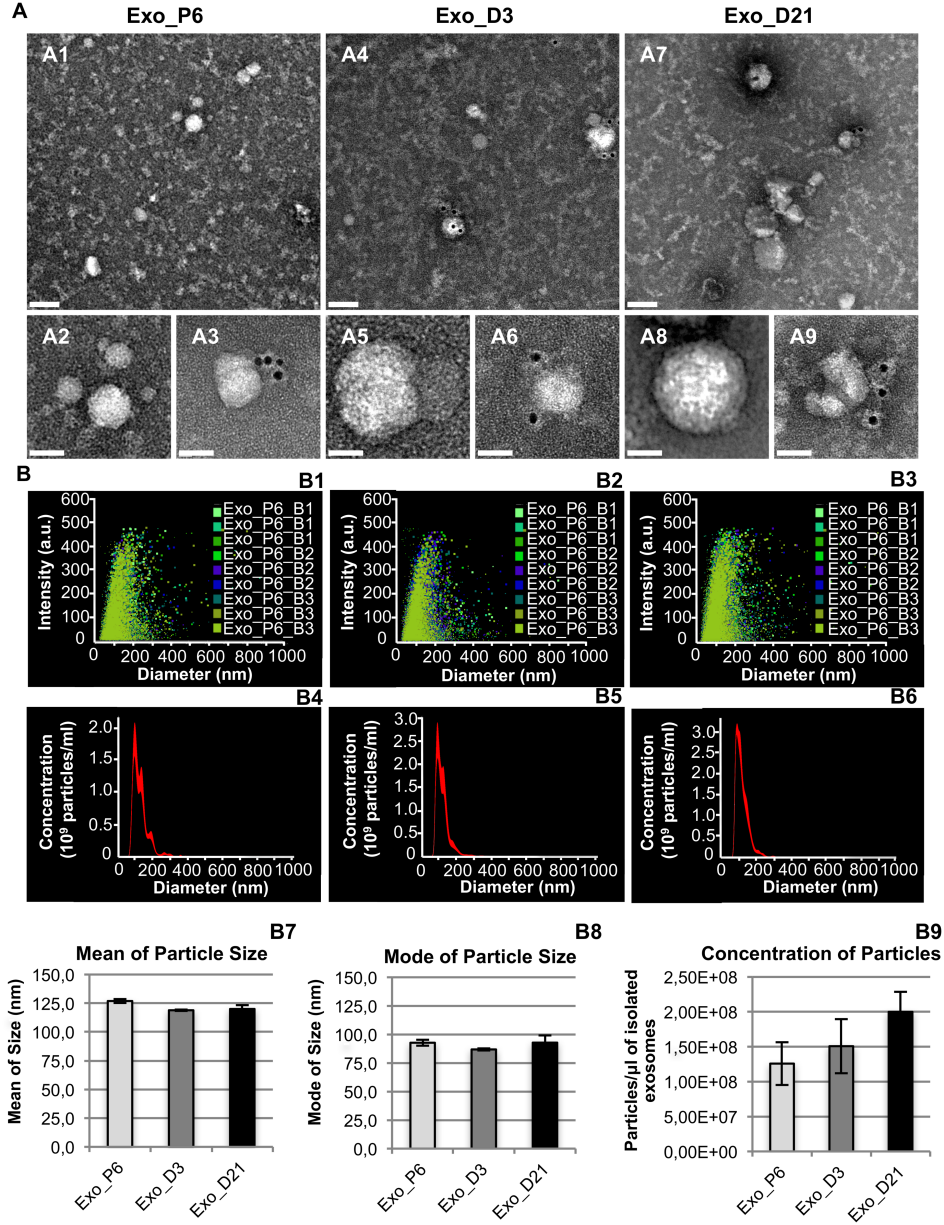
Characterisation of exosomes by transmission electron microscopy (A) and nanoparticle tracking analysis (B). (A) A1, A4 and A7: Survey transmission electron micrographs of exosomes from the Exo_P6, Exo_D3 and Exo_D21 groups. Exosomes are approximately 30–150 nm and mainly display a rounded shape. A4-A9: Details of selected exosomes without (A2, A5 and A8) or with (A3, A5 and A9) labelling of 10 nm gold-labelled anti-CD63 antibodies. Scale bar: top panel (A1, A4 and A7), 100 nm; bottom panel (A2-A3, A5-A6 and A8-A9), 50 nm. (B) B1-B3: Distribution of particles from all nine captures of three biological replicates in each group of exosomes. B4-B6: Average concentration/size distribution of all nine captures in each group of exosomes. B7-B8: Mean and mode of particle size of each group of exosomes. B9: Mean concentration of particles in each group of exosomes. Error bars in B7-B9 denote standard error mean, SEM; n = 3 biological replicates.

Exosomes were isolated from conditioned media of hMSCs expansion and different time points (D3, D6, D9, D12, D15, D18 and D21) of osteogenic differentiation through a series of ultrafiltration and ultracentrifugation. A schematic illustration of the experimental setup is shown in Fig 1. Exosomes from expansion (Exo_P6), early differentiation (Exo_D3) and late differentiation (Exo_D21) were selected as representative samples for exosome characterisation by transmission electron microscopy (TEM) and nanoparticle tracking analysis (NTA). Exosomes were immunolabelled with exosome marker CD63 and examined with TEM. The electronic micrographs revealed that all three groups of examined exosomes had rounded structures and a 30–150 nm size range (Fig 2A). Although some exosomes were labelled with one or multiple anti-CD63 conjugated gold particles, many of the exosomes were non-labelled. Collectively, the micrographs showed different groups of exosomes of similar shape and size range.

The selected exosome samples were diluted with PBS in a proper ratio and determined by NTA (Fig 2B). The distribution of nanoparticles from all nine video captures of each exosome group is shown in Fig 2B1–2B3. In general, the most intensive nanoparticle populations were distributed in a 30–150 nm size range. The average concentration and size distribution of nanoparticles are shown in Fig 2B4–2B6. The mean and mode size of nanoparticles in Exo_P6, Exo_D3 and Exo_D21 were 127 nm/93 nm, 119 nm/87 nm and 120 nm/93 nm respectively (Fig 2B7 and 2B8). The concentrations of nanoparticles in Exo_D3 (1.51 ± 0.39 x10^8^ /μl) and Exo_D21 (2.00 ± 0.29 x10^8^ /μl) were slightly increased compared with that in Exo_P6 (1.26 ± 0.31 x10^8^ /μl) (Fig 2B9). The statistical analysis revealed no significant difference in either the size or concentration of nanoparticles among the three groups of exosomes.

### Exosomes derived from hMSCs induce osteogenic differentiation of hMSCs in a stage-dependent manner

**Fig 3:**
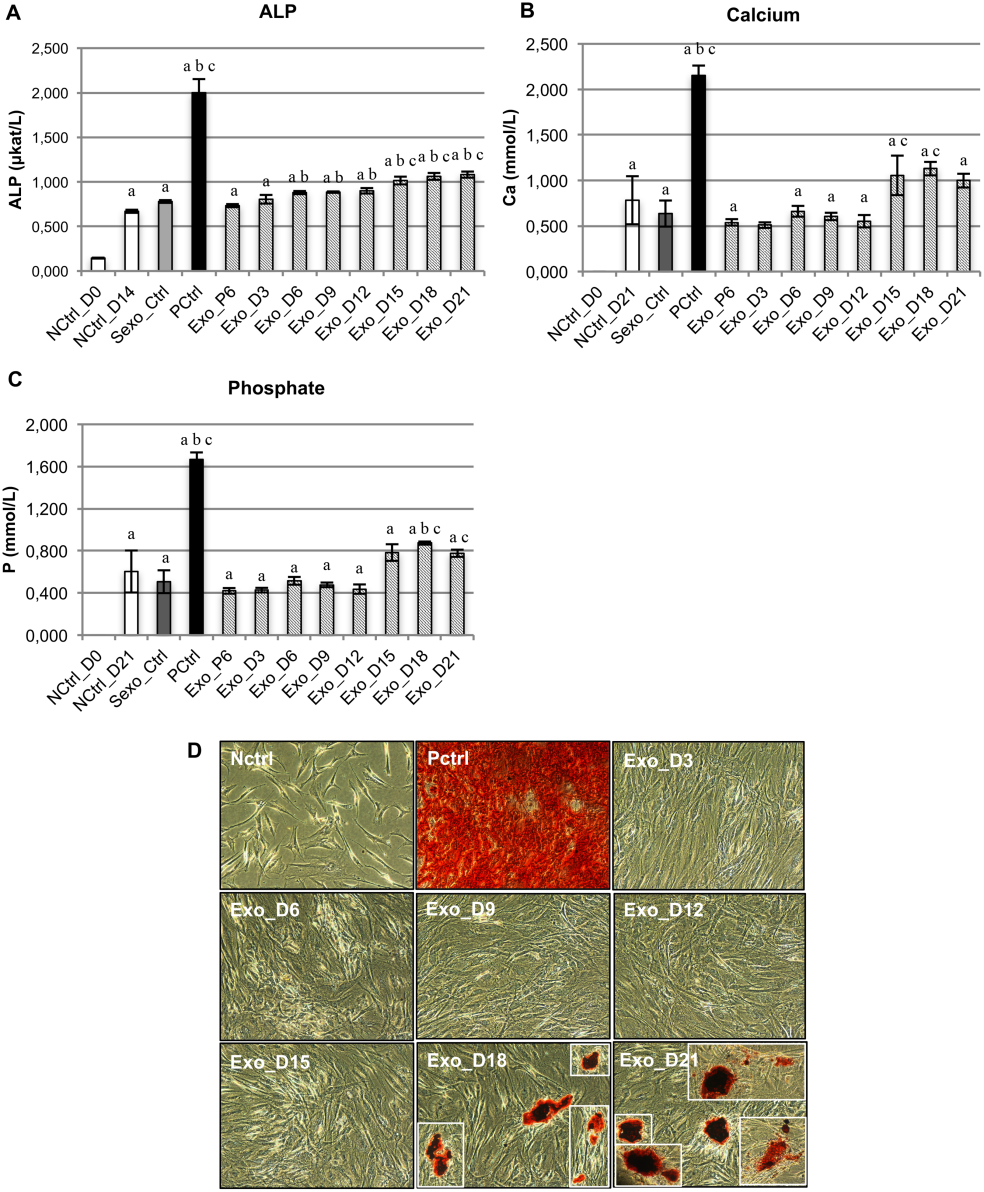
Evaluation of osteogenic differentiation of hMSCs after exosome treatment. (A) ALP activity 14 d after treatment with exosomes or the appropriate controls; (B) Quantification of calcium in ECM after 21 d; (C) Quantification of phosphate in ECM after 21 d. In (A-C), bars indicate mean values whereas error bars denote standard errors of the mean (SEM). Small letters a, b and c represent statistical significance when compared with NCtrl_D0, NCtrl_D14/D21 and Sexo_Ctrl respectively, based on Bonferroni-corrected p value < 0.05. n = 3 independent experiments; (D) Alizarin red staining after 21 d.

To explore the functions of exosomes derived from hMSCs undergoing expansion and osteogenic differentiation, specifically on osteogenic lineage commitment, exosomes from expansion and different stages of differentiation were applied as stimuli (without any other chemical osteogenic supplements) to treat undifferentiated homotypic MSCs. After 14 d of treatment, the ALP activity was determined as an indicator of cell differentiation towards the osteogenic lineage (Fig 3A). All experimental groups of the treated MSCs revealed a significant increase in ALP activity compared with the initial time point (NCtrl_D0). However, only exosomes from D6-D21 of osteogenic differentiation, and not from the expansion (Exo_P6) and early differentiation (Exo_D3), induced a significant increase in ALP activity compared with negative controls without any stimuli (NCtrl_D14). Similarly, in comparison to negative controls without MSC-derived exosomes (Sexo_Ctrl), only exosomes from the late stages of osteogenic differentiation (Exo_D15, Exo_D18 and Exo_D21) resulted in a significant increase in ALP activity.

After 21 d of treatment, we quantified the calcium and phosphate content to investigate the progress of osteogenic differentiation in the ECM. Due to the undetectable level of calcium and phosphate content at the initial time point (D0), all groups of treatments showed a significant increase in calcium and phosphate when compared with NCtrl_D0 (Fig 3B and 3C). However, only exosomes from the late stages of osteogenic differentiation (Exo_D15 and Exo_D18) were capable of inducing the significant production of calcium in the ECM when compared with Sexo_Ctrl. Moreover, only treatments with Exo_D18 and Exo_D21 had significantly increased phosphate levels compared with NCtrl_D21 and/or Sexo_Ctrl. In comparison with the standard chemical stimuli for osteogenic differentiation (PCtrl), the production of calcium and phosphate in ECM induced by exosomes from the late stage of osteogenic differentiation was about 50% of that in PCtrl and about 1.35 times that in NCtrl_D21 without any stimuli. The significant production of calcium content in the ECM under the stimulation of exosomes from the late stages of osteogenic differentiation was also confirmed by Alizarin Red staining (Fig 3D). After 21 d of treatment, only Exo_D18- and Exo_D21-treated cells showed positive staining, indicating calcium deposits in the ECM. Taken together, the present data demonstrated that exosomes derived from hMSCs from various stages of osteogenic differentiation were able to commit homotypic cells to differentiate towards the osteogenic lineage, but only exosomes from the late stages of osteogenic differentiation were able to induce the mineralisation of treated homotypic cells.

### hMSCs internalise exosomes derived from the expansion and osteogenic differentiation of hMSCs

**Fig 4:**
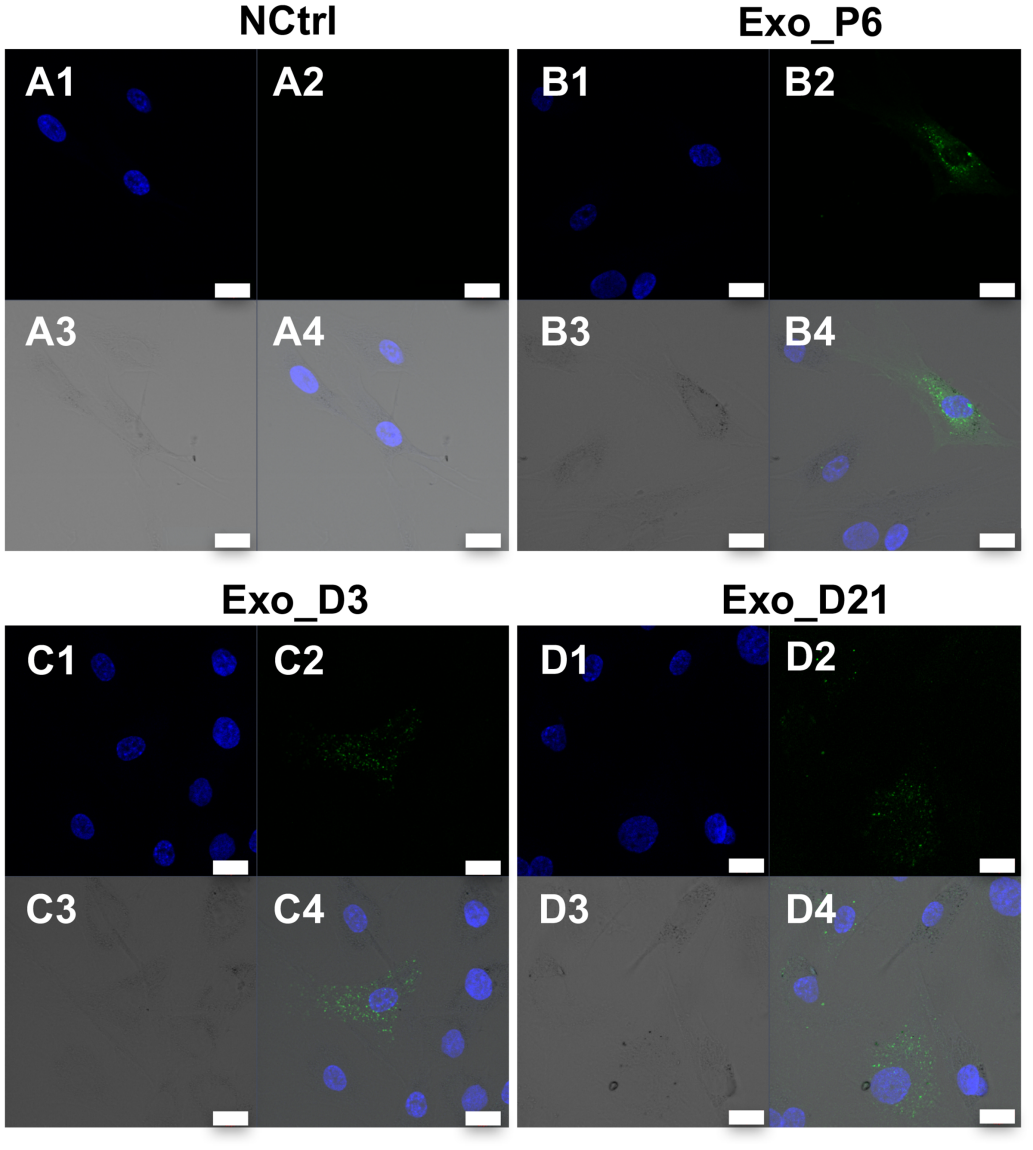
Internalisation of exosomes in hMSCs. Confocal micrographs of hMSCs incubated for 24h with A) PBS (negative control); B) Exo_P6; C) Exo_D3 and D) Exo_D21. PKH67-stained exosomes are detected mainly in the cytoplasm of some of the cells. The intensity varied between individual cells. No PKH67-stained material was found in the negative control. A1-D1, blue channel; A2-D2, green channel; A3-D3, transmission (TD) channel; A4-D4, merged channels. Blue, DAPI-stained nucleus; Green, PKH67-stained exosomes. Scale bar: 20 μm.

To examine whether hMSCs-derived exosomes interact with homotypic cells, three representative groups of exosomes, Exo_P6, Exo_D3 and Exo_D21, were labelled with green fluorescence dye PKH67 and incubated with hMSCs. After 24 h of incubation, all three groups of exosomes were internalised by hMSCs. The internalised PKH67-labelled exosomes were localised in the cytoplasm of hMSCs (Fig 4 and S1 Fig). Irrespective of the cell source of exosomes, confocal microscopy revealed that some cells were positive, with PKH67 staining, while other cells in the same culture were not, indicating that only a specific fraction of the treated cells were able to internalise PKH67-labelled exosomes. The intracellular localization of PKH67-labelled exosomes was clearly shown by Z-stack gallery and orthographic view (S1 Fig). The confocal images from in-depth to the surface showed that the intensity of PKH67 stained exosomes and DAPI stained nuclei increased or decreased in the same trend. The orthographic view showed exosomes and nuclei localized in the same focus depth, which further confirmed the internalisation of exosomes into hMSCs. Moreover, the PKH67 fluorescence intensity of positive cells in the same culture varied, suggesting that the amount of exosomes internalised by individual cells differed (Fig 4D).

### MicroRNA profiles of exosomes and hMSCs during expansion and osteogenic differentiation are altered

**Fig 5:**
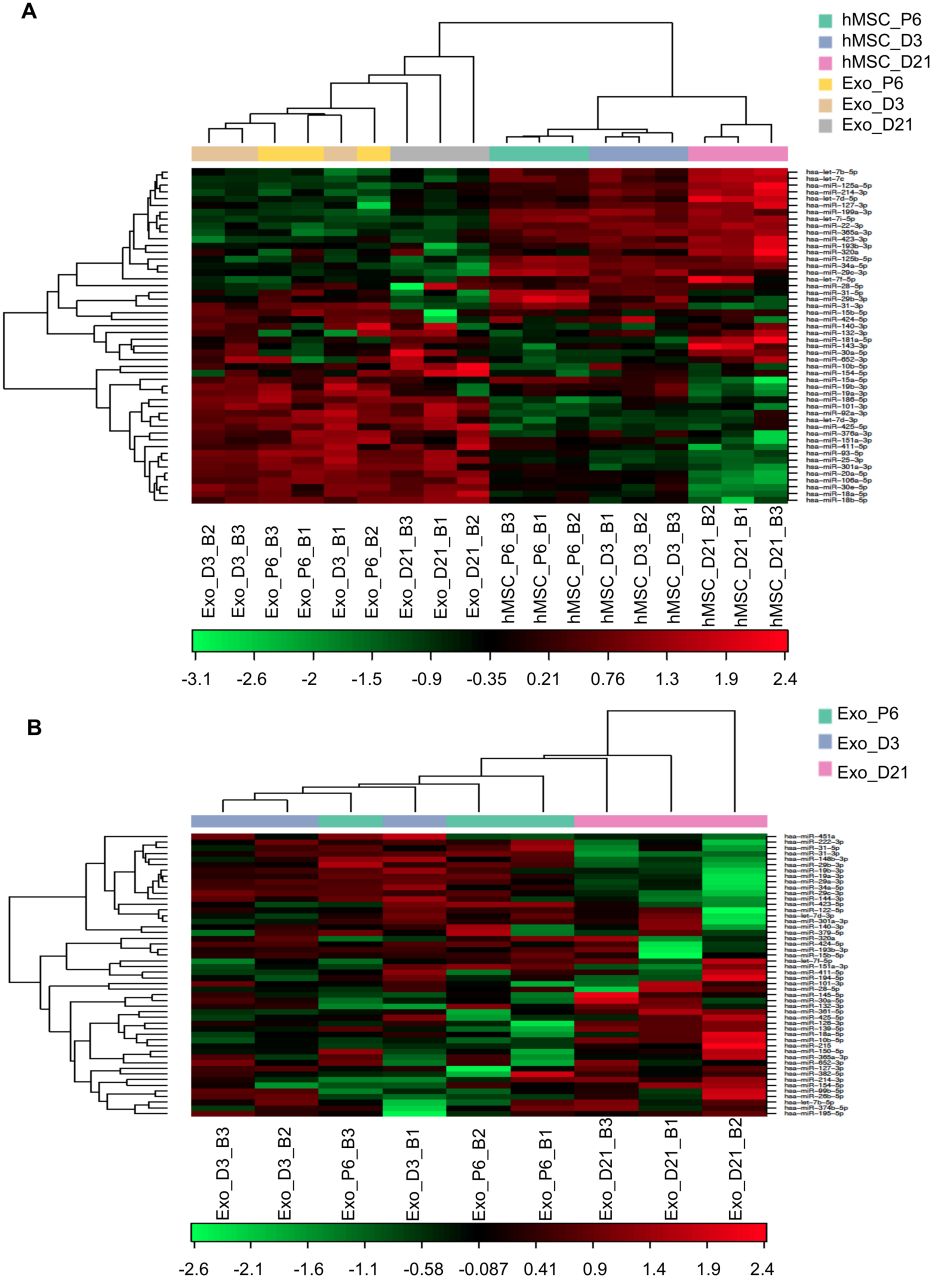
MicroRNA profiles of exosomes and hMSCs during expansion and osteogenic differentiation. (A) Altered microRNA profiles of exosomes and hMSCs during expansion (P6) and osteogenic differentiation (D3 and D21) of hMSCs and (B) Altered microRNA profiles of exosomes derived from the expansion (P6) and osteogenic differentiation (D3 and D21) of hMSCs. The heat map diagram shows the result of the two-way hierarchical clustering of microRNAs and samples. The clustering is performed on all samples and on the top 50 microRNAs with the highest standard deviation. The normalised (dCq) values have been used for the analysis. Each row represents one microRNA and each column represents one sample. The microRNA clustering tree is shown on the left. The colour scale shown at the bottom illustrates the relative expression level of a microRNA across all samples: red colour represents an expression level above the mean, green colour represents expression lower than the mean.

To investigate the content of exosomes that may be responsible for the osteogenic induction by exosomes, we profiled the microRNA expression of three representative groups of exosomes, Exo_P6, Exo_D3 and Exo_D21, and their parent cells. A total of 77 microRNAs were identified in all samples, with an average of 183 microRNAs detectable per sample (S1 Table). Hierarchical clustering was done in R using scripts from Bioconductor. The clustering was performed with Pearson correlation and average linkage clustering. Clustering analysis of the microRNA expression in the three groups of exosomes versus their respective parent cells revealed marked differences in microRNA expression between exosomes and their respective parent cells (Fig 5A).

The differentially expressed microRNAs among the three groups of exosomes were also identified. Ninety-one microRNAs were identified in all exosome samples and an average of 160 microRNAs were detectable per exosome sample (S1 Table). The heat map generated by the normalised dCq values of the top 50 microRNAs with the highest standard deviation revealed a similar pattern of microRNA expression in exosomes from hMSC expansion (Exo_P6) and early osteogenic differentiation (Exo_D3), while exosomes from the late stage of differentiation (Exo_D21) had a different microRNA expression in comparison with Exo_P6 and Exo_D3 (Fig 5B). Statistical analysis revealed that one, nine and 16 microRNAs had a significantly differential expression when comparing the microRNA expression in Exo_D3 with Exo_P6, Exo_D21 with Exo_P6 and Exo_D21 with Exo_D3 respectively (Table 1). Among the differentially expressed microRNAs in Exo_D21 versus Exo_P6, five microRNAs (miR-31-3p, miR-31-5p, miR-29b-3p, miR-148b-3p and miR-29c-3p) were decreased two-fold or more, while only one microRNA (miR-10b-5p) had more than a two-fold increase in expression. However, when comparing Exo_D21 with Exo_D3, three microRNAs (miR-31-3p, miR-144-3p and miR-29c-3p) were reduced more than two-fold and two microRNAs (miR-154-5p and miR-10b-5p) were increased more than two-fold. Moreover, five microRNAs (miR-31-3p, miR-29b-3p, miR-29c-3p, miR-10b-5p and miR-27b-3p) had significant changes in expression in Exo_D21 compared with their expression in both Exo_P6 and Exo_D3. The changes in the expression of miR-10b-5p, miR-27b-3p and miR-29c-3p in Exo_D21 compared with Exo_P6 and Exo_D3 respectively were similar. Interestingly, the two highest regulated microRNAs in Exo_D21 versus Exo_D3 and Exo_P6, miR-31-3p/5p and miR-10b-5p, are involved in osteogenic differentiation and cell recruitment [30, 31].

**Table 1:** Differentially expressed microRNAs in exosomes derived from hMSCs during expansion and osteogenic differentiation.

Exosomes	microRNA	Fold changes	p value
Exo_D3 vs Exo_P6	hsa-miR-484	-1.5	0.0332
Exo_D21 vs Exo_P6	hsa-miR-10b-5p	2.4	0.0213
	hsa-miR-27b-3p	1.7	0.0180
	hsa-miR-23b-3p	1.5	0.0309
	hsa-miR-31-3p	-6.2	0.0006
	hsa-miR-31-5p	-4.1	0.0490
	hsa-miR-29b-3p	-2.2	0.0073
	hsa-miR-148b-3p	-2.0	0.0251
	hsa-miR-29c-3p	-2.0	0.0368
	hsa-miR-221-3p	-1.6	0.0328
Exo_D21 vs Exo_D3	hsa-miR-154-5p	2.6	0.0483
	hsa-miR-10b-5p	2.1	0.0265
	hsa-miR-18b-5p	1.9	0.0284
	hsa-miR-139-5p	1.9	0.0059
	hsa-miR-361-5p	1.7	0.0281
	hsa-miR-27b-3p	1.6	0.0004
	hsa-miR-152	1.6	0.0097
	hsa-miR-26a-5p	1.4	0.0304
	hsa-miR-21-5p	1.4	0.0195
	hsa-miR-126-3p	1.4	0.0108
	hsa-miR-24-3p	1.4	0.0052
	hsa-miR-31-3p	-4.7	0.0035
	hsa-miR-144-3p	-2.3	0.0272
	hsa-miR-29c-3p	-2.1	0.0330
	hsa-miR-29b-3p	-1.6	0.0361
	hsa-miR-15a-5p	-1.6	0.0481

To further reveal the correlation of microRNA expression among the three groups of exosomes, and between exosomes and their respective parent cells, the top 50 abundant microRNAs in each sample were sorted based on the normalised dCq value (S2 Table). Interestingly, 42 microRNAs (84% of the sorted 50 microRNAs) were expressed in all three groups of exosomes. Among the remaining eight microRNAs in each top 50 lists, 6% appeared in both Exo_P6 and Exo_D3, equally 6% were shared between Exo_D3 and Exo_D21, whereas only 2% were present in both Exo_P6 and Exo_D21. In addition, 8%, 4% and 8% microRNAs were specifically present in the top 50 abundant microRNAs of Exo_P6, Exo_D3 and Exo_D21 respectively (S2 Fig and S3 Table). Moreover, the comparison of the top 50 abundant exosomal and cellular microRNAs between Exo_P6 versus hMSCs_P6, Exo_D3 versus hMSCs_D3 and Exo_D21 versus hMSC_D21 showed that 74%, 76% and 70% respectively of the sorted microRNAs were shared between exosomes and their respective parent cells (S2 Fig and S4 Table).

Taken together, the data showed altered microRNA profiles of exosomes and their respective parent cells during expansion and the early and late stages of osteogenic differentiation. Exosomal microRNAs were differentially expressed in the late stage of osteogenic differentiation compared with expansion and the early stage of differentiation. Comparisons of the top 50 abundant microRNAs in each sample revealed a high correlation of microRNA content between exosomes and their respective parent cells.

### Differentially expressed exosomal microRNAs enrich pathways related to osteogenic differentiation and general regulation mechanisms

In order to explore the functions of the differentially expressed exosomal microRNAs, their target genes and Kyoto Encyclopedia of Genes and Genomes (KEGG) biological pathways were analysed [32]. All significantly affected KEGG pathways, analysed via predicted interaction and experimentally validated interaction, by the three groups of differentially expressed microRNAs were summarised (Table 2 and S5–S7 Tables). Furthermore, the significantly enriched KEGG pathways related to osteogenic differentiation were sorted (Table 3). A total of three, twenty and twenty KEGG pathways directly or indirectly related to osteogenic differentiation were enriched by the differentially expressed microRNAs in the comparisons of Exo_D3 versus Exo_P6, Exo_D21 versus Exo_P6 and Exo_D21 versus Exo_D3 respectively. It is noteworthy that the enriched KEGG pathways included the Wnt, MAPK, Hippo, mTOR and FoxO signalling pathways, known to be involved in the regulation of osteogenic differentiation. The regulation of the Wnt signalling pathway, by differentially expressed microRNAs in Exo_D21, is shown in detail (S3 Fig). Although the differentially expressed microRNAs in Exo_D21 versus Exo_P6 and Exo_D21 versus Exo_D3 were different, the enriched KEGG pathways, regulating osteogenic differentiation, by both groups of microRNAs overlapped substantially. Among the total of significantly enriched KEGG pathways, pathways related to regulation mechanisms, which may provide possible mechanisms for the way exosomes exert their functions, were further sorted (Table 3). The differentially expressed microRNAs in Exo_D21 versus Exo_P6 and in Exo_D21 versus Exo_D3 both significantly enriched the pathways of endocytosis (hsa04144) and regulation of actin cytoskeleton (hsa04810), which are possible pathways regulating the internalisation of exosomes to recipient cells, thereby creating an opportunity for exosomes to exert their functions on the recipient cells. Moreover, enriched pathways Spliceosome (hsa03040), RNA transport (hsa03013), mRNA surveillance pathway (hsa03015) and protein digestion and absorption (hsa04974) may indicate how exosomal microRNAs regulate the functions of recipient cells.

**Table 2:** Summary of biological pathways enriched by differentially expressed microRNAs.

Comparison	Total number of differentially expressed microRNAs	Total number of enriched KEGG pathways (p-value < 0.05, FDR corrected)	
		microT-CDS	Tarbase
Exo_D3 vs Exo_P6	1	3	18
Exo_D21 vs Exo_P6	9	41	65
Exo_D21 vs Exo_D3	16	63	77

**Table 3:** Enriched KEGG biological pathways related to osteogenic differentiation and general regulation mechanism.

**Comparison**	**KEGG pathways related to osteogenic differentiation**		**p-value, FDR corrected**	**Genes**	**miRNAs**	**Algorithms**
Exo_D3 vs Exo_P6	Hippo signalling pathway	(hsa04390)	1.41E-05	9	1	microT-CDS
			4.21E-02	16	1	Tarbase
	Adherens junction	(hsa04520)	6.59E-03	6	1	microT-CDS
			3.98E-04	15	1	Tarbase
	ECM-receptor interaction	(hsa04512)	5.63E-05	11	1	Tarbase
Exo_D21 vs Exo_P6	ECM-receptor interaction	(hsa04512)	8.84E-63	40	8	microT-CDS
		(mmu04512)	8.27E-07	36	9	Tarbase
	Focal adhesion	(hsa04510)	7.16E-07	79	9	microT-CDS
		(mmu04510)	1.53E-05	110	9	Tarbase
	Estrogen signalling pathway	(hsa04915)	1.30E-03	32	9	microT-CDS
			1.38E-05	54	9	Tarbase
	Signalling pathways regulating pluripotency of stem cells	(hsa04550)	2.66E-03	46	9	microT-CDS
			1.38E-05	73	9	Tarbase
	FoxO signalling pathway	(hsa04068)	1.49E-02	44	8	microT-CDS
			2.37E-04	74	9	Tarbase
	Wnt signalling pathway	(hsa04310)	2.23E-02	41	9	microT-CDS
			8.51E-03	66	9	Tarbase
	mTOR signalling pathway	(hsa04150)	2.65E-02	23	7	microT-CDS
			1.90E-04	38	9	Tarbase
	Hippo signalling pathway	(hsa04390)	4.14E-02	37	9	microT-CDS
			1.35E-07	74	8	Tarbase
	Ras signalling pathway	(hsa04014)	2.27E-03	65	9	microT-CDS
	Rap1 signalling pathway	(hsa04015)	4.48E-03	65	9	microT-CDS
	MAPK signalling pathway	(hsa04010)	1.85E-02	74	9	microT-CDS
	cAMP signalling pathway	(hsa04024)	4.14E-02	58	9	microT-CDS
	PI3K-Akt signalling pathway	(hsa04151)	2.12E-04	104	9	microT-CDS
	TGF-beta signalling pathway	(hsa04350)	1.35E-07	45	9	Tarbase
	Adherens junction	(hsa04520)	3.29E-07	45	9	Tarbase
	TNF signalling pathway	(hsa04668)	2.05E-03	55	9	Tarbase
	HIF-1 signalling pathway	(hsa04066)	3.13E-03	56	9	Tarbase
	AMPK signalling pathway	(hsa04152)	5.94E-03	62	9	Tarbase
	Insulin signalling pathway	(hsa04910)	2.29E-02	67	9	Tarbase
	VEGF signalling pathway	(hsa04370)	3.66E-02	32	9	Tarbase
Exo_D21 vs Exo_D3	ECM-receptor interaction	(hsa04512)	7.57E-35	44	14	microT-CDS
		(mmu04512)	8.26E-08	47	15	Tarbase
	Signalling pathways regulating pluripotency of stem cells	(hsa04550)	4.86E-10	75	16	microT-CDS
			3.29E-05	86	15	Tarbase
	Hippo signalling pathway	(hsa04390)	1.11E-08	73	15	microT-CDS
		(rno04390)	7.86E-08	88	14	Tarbase
	Focal adhesion	(hsa04510)	4.67E-08	107	17	microT-CDS
		(rno04510)	3.94E-06	130	15	Tarbase
	PI3K-Akt signalling pathway	(hsa04151)	1.68E-07	155	16	microT-CDS
		(rno04151)	3.54E-04	178	15	Tarbase
	TGF-beta signalling pathway	(hsa04350)	1.67E-05	42	12	microT-CDS
			1.05E-07	53	14	Tarbase
	mTOR signalling pathway	(hsa04150)	1.67E-05	37	15	microT-CDS
		(rno04150)	1.29E-02	40	15	Tarbase
	MAPK signalling pathway	(hsa04010)	1.67E-05	118	16	microT-CDS
			6.21E-03	137	14	Tarbase
	FoxO signalling pathway	(hsa04068)	3.23E-05	67	16	microT-CDS
		(rno04068)	5.86E-07	92	14	Tarbase
	AMPK signalling pathway	(hsa04152)	1.31E-04	62	15	microT-CDS
		(rno04152)	8.95E-03	75	15	Tarbase
	Estrogen signalling pathway	(hsa04915)	1.06E-03	43	15	microT-CDS
			4.71E-04	61	14	Tarbase
	Insulin signalling pathway	(hsa04910)	2.83E-02	59	15	microT-CDS
			5.32E-05	89	15	Tarbase
	Wnt signalling pathway	(hsa04310)	4.85E-06	68	15	microT-CDS
	Ras signalling pathway	(hsa04014)	2.74E-07	104	16	microT-CDS
	Rap1 signalling pathway	(hsa04015)	2.06E-04	94	15	microT-CDS
	cAMP signalling pathway	(hsa04024)	7.09E-04	88	15	microT-CDS
	cGMP-PKG signalling pathway	(hsa04022)	2.92E-02	68	16	microT-CDS
	Adherens junction	(hsa04520)	7.00E-07	52	14	Tarbase
	TNF signalling pathway	(hsa04668)	1.08E-05	73	15	Tarbase
	HIF-1 signalling pathway	(hsa04066)	2.96E-03	66	15	Tarbase
**Comparison**	**KEGG pathways related to general regulation mechanism**		**p-value, FDR corrected**	**Genes**	**miRNAs**	**Algorithms**
Exo_D3 vs Exo_P6	Spliceosome	(hsa03040)	4.21E-02	22	1	Tarbase
Exo_D21 vs Exo_P6	Protein digestion and absorption	(hsa04974)	1.51E-02	32	8	microT-CDS
	Regulation of actin cytoskeleton	(hsa04810)	3.92E-02	65	9	microT-CDS
	Endocytosis	(hsa04144)	2.69E-02	96	9	Tarbase
	mRNA surveillance pathway	(hsa03015)	3.95E-02	47	9	Tarbase
Exo_D21 vs Exo_D3	Regulation of actin cytoskeleton	(hsa04810)	5.08E-03	93	17	microT-CDS
			6.36E-03	116	15	Tarbase
	Protein digestion and absorption	(hsa04974)	2.92E-02	40	12	microT-CDS
	Endocytosis	(hsa04144)	1.48E-04	121	15	Tarbase
	RNA transport	(hsa03013)	1.44E-03	97	15	Tarbase
	Spliceosome	(hsa03040)	4.72E-02	71	14	Tarbase

Taken together, these results suggested that the differentially expressed exosomal microRNAs were able to regulate pathways involved in osteogenic differentiation and possible mechanisms through which exosomes exert their biological functions.

## Discussion

A growing body of studies have demonstrated therapeutic effects by MSC-derived exosomes on wound healing and tissue repair in various physiological systems [33], which has led to great interest in the utilisation of MSC-derived exosomes in tissue engineering and regenerative medicine [12]. It has been suggested that MSC-derived exosomes mediate their functions on tissue repair/regeneration via the promotion of angiogenesis [34–36]. Moreover, MSC-derived exosomes possess immunomodulatory potential and are capable of attenuating inflammation [34, 37]. Overall, current studies indicate that MSC-derived exosomes at least partially mimic the properties and function of their parent cells.

Exosomes are secreted by MSCs undergoing early osteogenic differentiation [38, 39], but the secretion and function of exosomes during the entire osteogenic differentiation process of MSCs has not been investigated. Less attention has been focused on the effects of MSC-derived exosomes on the induction of differentiation, which is recognised as one of the most important properties of MSCs during tissue regeneration. Furthermore, the underlying mechanism by which the secreted exosomes may exert their function has not been described. In the present study, we observed that MSCs secreted exosomes through the entire process of expansion and osteogenic differentiation and that a specific population of recipient MSCs internalised MSC-derived exosomes. Moreover, the MSC-derived exosomes induced osteogenic differentiation and mineralisation in a stage-dependent manner. Further profiling of the exosomal microRNA content and bioinformatic analysis of their target genes and cell signalling pathways indicate that exosomal microRNAs are at least partly responsible for the observed effects.

Previous studies have demonstrated that the osteogenic differentiation of MSCs is promoted by conditioned media from monocytes [40], exosomes derived from monocytes [28] and MSCs undergoing early osteogenic differentiation [39]. Further, factors secreted by MSCs during osteogenic differentiation promote bone formation *in vivo* [41, 42]. The present observation that MSC-derived exosomes significantly increased ALP activity and ECM mineralisation in a stage-dependent manner suggests that the osteoinductive effect of MSCs observed in previous studies might be partially mediated by MSC-derived exosomes. The mechanism by which exosomes induce osteogenic differentiation and mineralisation is not clear. A recent review suggested that exosomes and matrix vesicles, unique extracellular membrane-bound microparticles serving as initial sites for mineral formation, are homologous structures through an analysis of size, morphology and lipid and protein content [43]. After release from cells, exosomes may anchor to extracellular matrix and adopt the morphological appearance and functional activities of matrix vesicles. Nevertheless, more studies need to be conducted to investigate how exosomes interact with extracellular matrix and serve as sites for mineralisation.

Despite the interaction with extracellular matrix by surface protein, exosomes may exert their function through internalisation into cells. Exosomes have been shown to mediate cell-to-cell communication in the absence of direct cell-to-cell contact. To further understand how MSC-derived exosomes induced the observed effects, we examined whether these exosomes could be internalised into homotypic cells. We observed that only a subpopulation of MSCs internalised PKH67-labelled exosomes. This may be due to the heterogeneity of MSCs in terms of their surface receptors, as well as the different phase of the cell cycle. In addition, although exosomes share a similar size, flotation density in a sucrose gradient and possess some common exosome-associated protein markers such as CD63, CD9 and CD81, it has been suggested that exosomes may contain various surface receptors or ligands, which are able to activate receptor-dependent signalling pathways to mediate their internalisation [44, 45]. In fact, the qualitative TEM observations revealed that only a subpopulation of MSC-derived exosomes was labelled with anti-CD63, which may provide evidence of variation among exosomes and their internalisation by recipient cells due to the possible roles of tetraspanins in the internalisation of exosomes [46]. Exosomes have been found to be internalised via membrane fusion or different endocytic pathways, including clathrin-dependent endocytosis, caveolae-dependent endocytosis, lipid raft-mediated endocytosis, macropinocytosis and phagocytosis [46]. Interestingly, pathways of “endocytosis” and “regulation of actin cytoskeleton” were enriched by differentially expressed exosomal microRNAs from the late stage of differentiation. This result indicates that exosomal microRNAs play a role in the regulation of exosome internalisation. However, it still remains for future investigations to address questions such as which distinguished population of MSCs is able to internalise exosomes, which exosomal receptors/ligands activate the pathways for their internalisation and how exosomal microRNAs influence the internalisation of exosomes.

It has been suggested that MSC exosomes mediate their therapeutic effects in a content-dependent manner, in which MSC exosomes were shown to function via proteins, mRNAs and/or microRNAs [47]. Therapeutic effect mediated by MSC-derived exosomal protein was shown in several studies [5, 35, 48, 49], where MSC-derived exosomes exerted their function via the enzymatic activity of exosomal proteins e.g. Wnt4 [35, 49] and neprilysin [48]. Therefore, the protein, mRNA and other contents of exosomes are of importance for the biological effects mediated by exosomes. However, in the present study, we mainly focused on studying the microRNA contents of exosomes. Recently, several studies have been conducted to determine exosomal microRNA-mediated therapeutic effects on tissue repair/regeneration [19, 23, 50, 51]. In the present study, due to the limitation of exosomal RNA amount, a selected commercially available panel of microRNA candidates was profiled. However, exosomes may also contain microRNAs not included in this panel. Exosomes derived from MSCs in expansion and the early and late stages of osteogenic differentiation showed altered microRNA profiles and a set of microRNAs was differentially expressed in exosomes derived from MSCs in the late stage of differentiation compared with exosomes from expansion and the early stage of differentiation. Furthermore, the target prediction of the differentially expressed exosomal microRNAs showed significantly enriched signalling pathways regulating osteogenic differentiation. It is worth noting that, among the differentially expressed exosomal microRNAs, miR-31 was dramatically decreased in exosomes from the late stage of osteogenic differentiation. Interestingly, miR-31 has been shown to function as a negative regulator of osteogenesis and was down-regulated in MSCs during osteogenic differentiation [15, 31, 52–54]. MiR-31 has been reported to inhibit osteogenic differentiation via the down regulation of osteogenic transcription factors Osterix (Osx) and special AT-rich sequence-binding protein 2 (SATB2), which are both downstream of RUNX2 [15, 52]. The inhibition of miR-31 in MSCs improves the repair of bone defects *in vivo* indicated by increased bone volume and bone mineral density [31, 53]. Moreover, microvesicular miR-31 derived from senescent endothelial cells inhibits the osteogenic differentiation of MSCs by down-regulating receptors of Wnt protein FZD3 [55]. This provides evidence that exosomal miR-31 is biologically active and can be transferred to the recipient cells to exert their function. Furthermore, in the present study, two other negative regulators of osteogenesis, miR-221 and miR-144, were also decreased in exosomes from the late stage of osteogenic differentiation. The genetic inhibition of miR-221 triggers the osteogenic differentiation of MSCs [56]. Moreover, conditioned medium from MSCs reduced miR-221 expression, which in turn up-regulated the expression of intercellular adhesion molecule-1 and resulted in the increased migration and adhesion of MSCs, leading to the enhanced healing of bone defects *in vivo* [57]. A recent study showed that miR-144 directly targeted Smad4 to inhibit the osteogenic differentiation of MSCs [58]. Taken together, the lower expression of exosomal miR-31, miR-221 and miR-144 from the late stage of differentiation may contribute to the induction of osteogenic differentiation, whereas the lower osteogenic differentiation of cells treated by exosomes from expansion or early osteogenic differentiation may partly be due to the delivery of higher exosomal miR-31, miR-221 and miR-144.

In addition to the negative regulators, several positive regulators of osteogenic differentiation, such as miR-21, also existed in the differentially expressed exosomal microRNAs. In consistency with the up-regulation of cellular miR-21 in MSCs during osteogenic differentiation, the exosomal miR-21 from the late stage of differentiation was significantly increased. MiR-21 was reported to target the inhibitor of osteogenic differentiation, Spry1, and the pluripotency marker, Sox2, resulting in osteogenic lineage commitment and the promotion of osteogenic differentiation [59, 60]. Moreover, miR-21 regulates the PI3K-AKT-GSK3β pathway, leading to the stabilisation and accumulation of β-catenin in the cytoplasm to activate the transcription of RUNX2 and finally an increase in the osteogenesis of MSCs [61]. The positive effects of miR-21 on osteogenic differentiation have been confirmed in *in-vivo* models showing enhanced bone formation in osteoporosis and accelerated bone fracture healing [59, 62, 63]. On the other hand, miR-10b, one of the most increased exosomal microRNAs in the late stage of differentiation, has not been reported directly to regulate osteogenic differentiation. However, a recent study revealed that the expression level of circulating miR-10b was increased in serum from patients after recent osteoporotic fracture compared with controls, indicating a potential function of miR-10b in relation to bone regeneration [18]. Moreover, miR-10b has been shown to promote the migration of MSCs, which may contribute to bone regeneration [30]. Taken together, it is suggested that the increased expressed exosomal microRNAs from the late stage of osteogenic differentiation are highly involved in the positive regulation of the osteogenic differentiation of the recipient MSCs. However, it still remains to validate the functions of these differentially expressed exosomal microRNAs in future studies.

Due to the critical regulatory roles of microRNAs on bone development and regeneration, microRNAs have been applied for bone regeneration, using two main strategies. In order to overcome the poor stability and limited ability of cellular uptake, the delivery of microRNAs requires vehicles that are able to protect them and at the same time efficiently transfer them through the cell membrane. The genetic modification of MSCs by the transfection of virus vectors containing specific microRNAs, resulting in the overexpression of pro-osteogenic microRNAs or the inhibition of anti-osteogenic microRNAs, has been found to accelerate osteogenic differentiation [56, 62, 63]. Furthermore, the combination of genetically modified MSCs and implant/scaffold has been shown to improve osteogenic differentiation and bone regeneration both *in vitro* and *in vivo* [31, 53, 64]. However, the virus vectors may induce potential toxicity and immunogenicity, which limits the clinical application of virus vector-transfected MSCs. These observations indicate the need for non-viral vectors for the microRNA transfection of MSCs. A recent study reported that chitosan/tripolyphosphate/hyaluronic acid nanoparticles may serve as a non-viral vector efficiently to deliver microRNAs to transfect MSCs, resulting in the significantly enhanced osteogenesis of MSCs [65]. In addition to using genetically modified MSCs as a vehicle for microRNA, the encapsulation of microRNAs into artificial nanoparticles directly immobilised on implant surfaces has also been found successfully to promote the osteogenic differentiation of MSCs [66, 67]. Collectively, MSC-mediated cell therapy, microRNA-mediated gene therapy and the combination of both appear promising for bone regeneration. Nevertheless, many challenges still remain. For example, genetically modified MSC therapy may increase the risk of tumorigenesis; artificial nanoparticles delivering microRNAs may be not efficiently internalised by recipient cells and only monotypic microRNA is usually encapsulated. The development of MSC-derived exosomes as a new therapy for bone regeneration may overcome some of the problems addressed above and at the same time offer several advantages. MSC-derived exosomes are naturally secreted by cells and mimic the properties of the cell membrane and they might therefore be more efficiently internalised by cells than artificial nanoparticles. Moreover, MSC-derived exosomes contain a cocktail of bioactive molecules, such as microRNAs, which may be able to regulate multiple targets at the same time and thus increase the therapeutic effects. The preconditioning of MSCs, such as osteogenic induction, creates opportunities to generate exosomes with specific functions.

In the present study, we isolated and characterized exosomes derived from MSCs using the state-of-art techniques, and further investigated the biological effects of exosomes secreted under different stages of osteogenic differentiation. To reveal the mechanism of the stage-dependent effects of exosomes on regulation of osteogenic differentiation that we observed, we further profiled and compared the microRNA contents of exosomes. Furthermore, we predicted the mRNA targets and signalling pathways that are regulated by the differentially expressed exosomal microRNAs by bioinformatics software and literature study. In line with other studies, we found that exosomes carried osteogenesis related microRNAs [68], which may be partially responsible for the observation of osteogenic induction by exosomes. However, the limitation of the present study is that we did not experimentally verify how such effects are regulated via exosomal microRNAs, specifically the differentially expressed microRNAs related to osteogenesis. Although this was beyond the scope of the present study, it is of interest in future studies to investigate how microRNAs exert their function via exosomes. Strategies to either endogenously or exogenously load specific microRNA into exosomes and further evaluate the effects of these modified exosomes can be applied to carry out such aims [25, 69].

## Conclusions

In conclusion, the present study shows that exosomes derived from hMSCs at the late stages of osteogenic differentiation can induce the osteogenic differentiation and mineralisation of recipient homotypic MSCs. MSC-derived exosomes were internalized by a subpopulation of MSCs. Further, microRNA profiling revealed a change in the expression of microRNAs in exosomes and their parent cells. Differentially expressed exosomal microRNAs from the late stages of osteogenic differentiation are osteogenesis related and enriched pathways related to osteogenic differentiation and mechanisms by which exosomes exert their functions. These results will deepen our understanding of the functions of MSC-derived exosomes and further benefit the application of MSC-derived exosomes for bone regeneration.

## Supporting information

S1 Fig Internalisation of PKH67-labelled exosomes in hMSCs. The Z-stack gallery (A) and orthographic view (B) show the intracellular localization of PKH67-labelled exosomes.
(PDF)

S2 Fig Correlation of top 50 abundant microRNAs (based on normalized dcq value) in exosomes and hMSCs. The pie charts show the correlation of top abundant microRNAs among the three groups of exosomes (A) and between exosomes and hMSCs (B-D). The number and percentage of microRNAs are both shown in the figure.
(PDF)

S3 Fig Schematic diagram of Wnt signalling pathway enriched by differentially expressed microRNAs in Exo_D21 compared with Exo_P6 and Exo_D3. microRNAs, predicted and/or experimentally validated to participate in Wnt signalling pathway through regulating their potential target genes, are marked in red. The schematic Wnt signalling pathway was adopted from KEGG database (Kanehisa, M., *et al*., *Nucleic Acids Res*, 2017) and the analysis of microRNAs (in red) participating in the pathway was analysed using DIANA-mirPath v.3.
(PDF)

S1 Table microRNA expression in MSCs and exosomes during expansion (P6), early (D3) and late (D21) osteogenic differentiation. B1, B2, and B3 indicate 3 biological repeats. The total number of samples that present each individual microRNA is summarised in the column “Count”. ND denotes not detected and BF denotes background filtered.
(PDF)

S2 Table Top 50 expressed microRNAs based on mean of normalized dcq value. (PDF)

S3 Table Correlation of top 50 expressed microRNAs among exosomes. (PDF)

S4 Table Correlation of top 50 expressed microRNAs between exosomes and hMSCs. (PDF)

S5 Table Biological pathways enriched by differentially expressed microRNAs in Exo_D3 vs Exo_P6. (PDF)

S6 Table Biological pathways enriched by differentially expressed microRNAs in Exo_D21 vs Exo_P6. (PDF)

S7 Table Biological pathways enriched by differentially expressed microRNAs in Exo_D21 vs Exo_D3. (PDF)
