# Supplementary material for: Mesenchymal stem cell-derived exosomes have altered microRNA profiles and induce osteogenic differentiation depending on the stage of differentiation
Source: PLoS One. 2018 Feb 15;13(2):e0193059. doi: 10.1371/journal.pone.0193059 (PMC5814093; doi:10.1371/journal.pone.0193059)
Supplement: S1 Fig — The Z-stack gallery (A) and orthographic view (B) show the intracellular localization of PKH67-labelled exosomes. (PDF) [file pone.0193059.s001.pdf]

Supplementary data

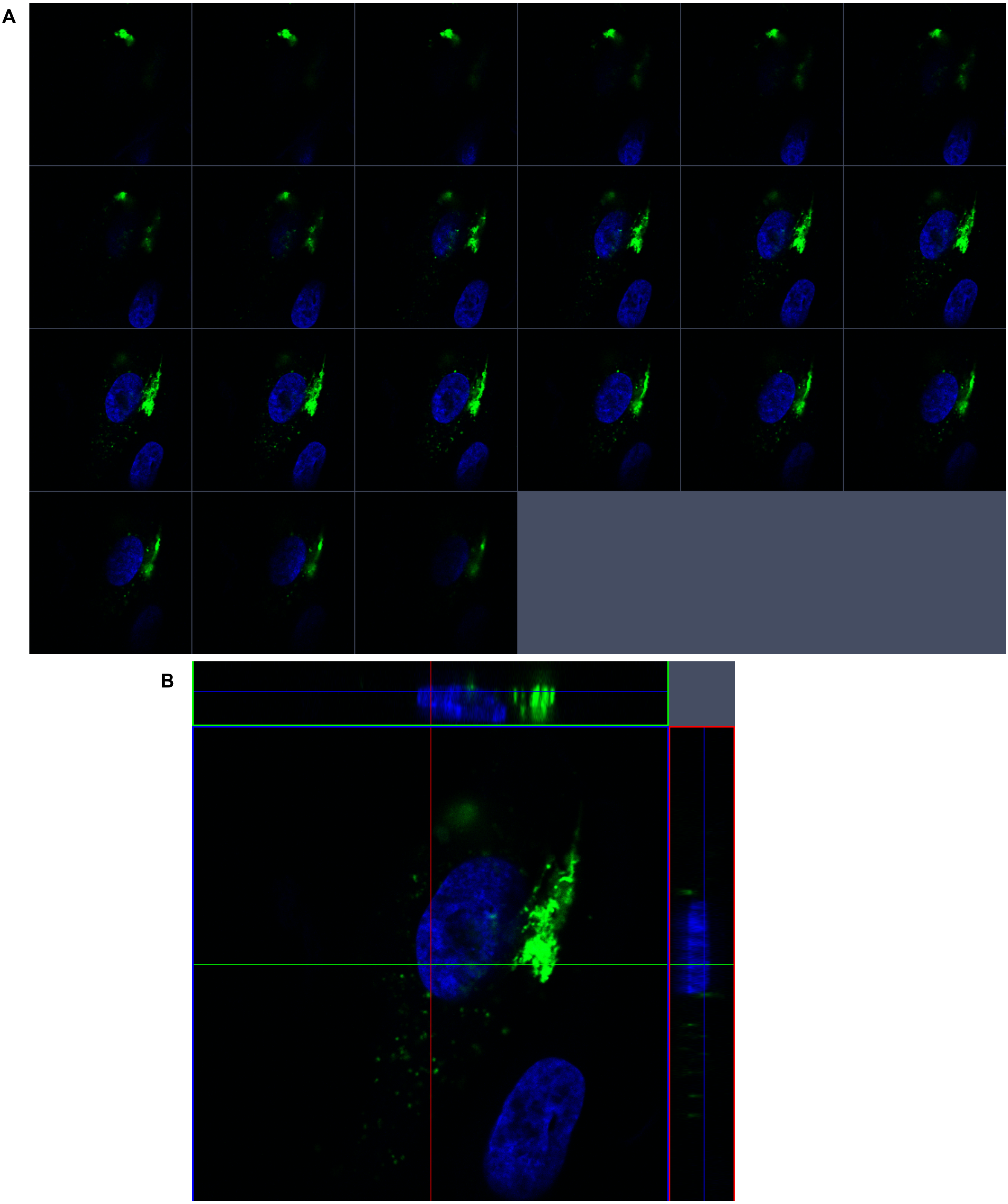

S1 Fig. Internalisation of PKH67-labelled exosomes in hMSCs.
The Z-stack gallery (A) and orthographic view (B) show the intracellular localization of PKH67-labelled exosomes.
