# Supplementary material for: Mesenchymal stem cell-derived exosomes have altered microRNA profiles and induce osteogenic differentiation depending on the stage of differentiation
Source: PLoS One. 2018 Feb 15;13(2):e0193059. doi: 10.1371/journal.pone.0193059 (PMC5814093; doi:10.1371/journal.pone.0193059)
Supplement: S2 Fig — The pie charts show the correlation of top abundant microRNAs among the three groups of exosomes (A) and between exosomes and hMSCs (B-D). The number and percentage of microRNAs are both shown in the figure. (PDF) [file pone.0193059.s002.pdf]

Supplementary data

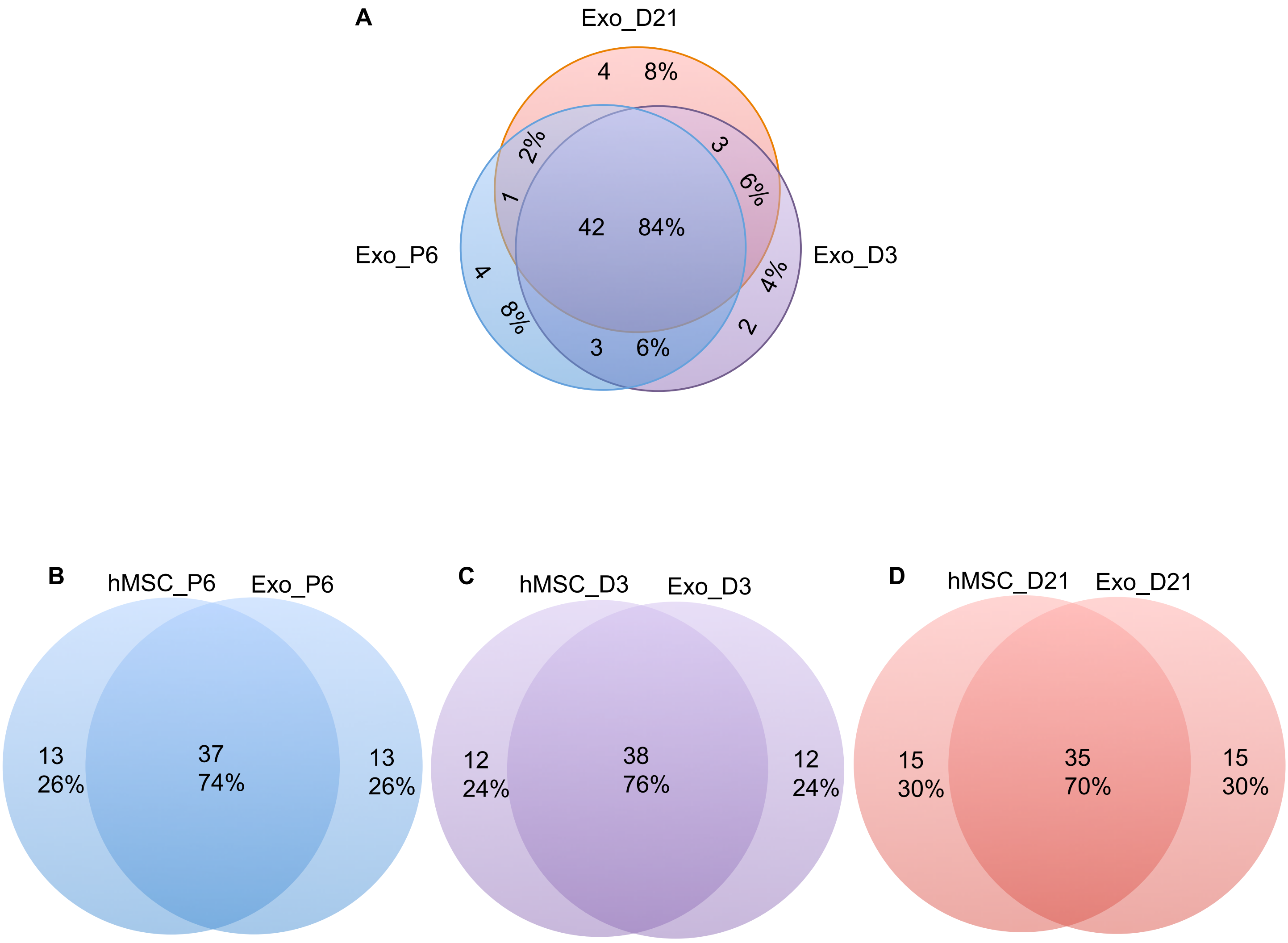

S2 Fig. Correlation of top 50 abundant microRNAs (based on normalized dcq value) in exosomes and hMSCs.

The pie charts show the correlation of top abundant microRNAs among the three groups of exosomes (A) and between exosomes and hMSCs (B-D). The number and percentage of microRNAs are both shown in the figure.
