# Supplementary material for: Mesenchymal stem cell-derived exosomes have altered microRNA profiles and induce osteogenic differentiation depending on the stage of differentiation
Source: PLoS One. 2018 Feb 15;13(2):e0193059. doi: 10.1371/journal.pone.0193059 (PMC5814093; doi:10.1371/journal.pone.0193059)
Supplement: S3 Fig — microRNAs, predicted and/or experimentally validated to participate in Wnt signalling pathway through regulating their potential target genes, are marked in red. The schematic Wnt signalling pathway was adopted from KEGG database (Kanehisa, M., et al., Nucleic Acids Res, 2017) and the analysis of microRNAs (in red) participating in the pathway was analysed using DIANA-mirPath v.3. (PDF) [file pone.0193059.s003.pdf]

Supplementary data

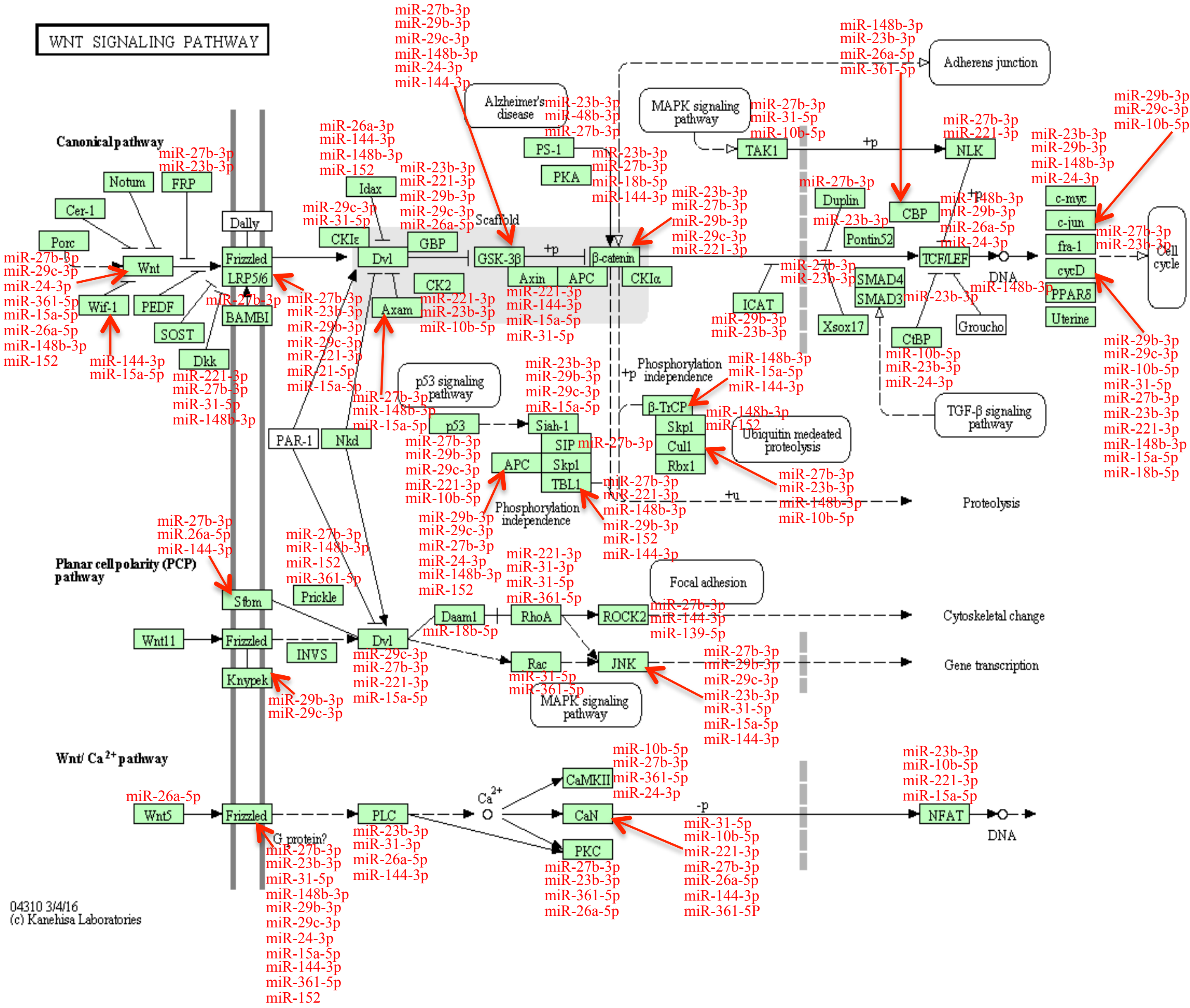

S3 Fig. Schematic diagram of Wnt signaling pathway enriched by differentially expressed microRNAs in Exo_D21 compared with Exo_P6 and Exo_D3. microRNAs, predicted and/or experimentally validated to participate in Wnt signaling pathway through regulating their potential target genes, are marked in red. The schematic Wnt signalling pathway was adopted from KEGG database (Kanehisa, M., *et al.*, *Nucleic Acids Res*, 2017) and the analysis of microRNAs (in red) participating in the pathway was analyzed using DIANA-mirPath v.3.
