# Supplementary material for: Mesenchymal stem cell-derived exosomes have altered microRNA profiles and induce osteogenic differentiation depending on the stage of differentiation
Source: PLoS One. 2018 Feb 15;13(2):e0193059. doi: 10.1371/journal.pone.0193059 (PMC5814093; doi:10.1371/journal.pone.0193059)
Supplement: S1 Table — B1, B2, and B3 indicate 3 biological repeats. The total number of samples that present each individual microRNA is summarised in the column “Count”. ND denotes not detected and BF denotes background filtered. (PDF) [file pone.0193059.s004.pdf]

Supplementary data

S1 Table. microRNA expression in MSCs and exosomes during expansion (P6), early (D3) and late (D21) osteogenic differentiation. B1, B2 and B3 indicate 3 biological repeats. The total number of samples that present each individual microRNA is summarised in the column “Count”. ND denotes not detected and BF denotes background filtered.

	MSC_P6_B1	MSC_P6_B2	MSC_P6_B3	MSC_D3_B1	MSC_D3_B2	MSC_D3_B3	MSC_D21_B1	MSC_D21_B2	MSC_D21_B3	exo_P6_B1	exo_P6_B2	exo_P6_B3	exo_D3_B1	exo_D3_B2	exo_D3_B3	exo_D21_B1	exo_D21_B2	exo_D21_B3	Count	Standard deviation
**hsa-miR-34a-5p**	25.3	25.6	25.5	26.3	25.9	26.2	26.6	26.5	27.3	32.8	33.5	31.0	31.6	30.9	29.9	34.0	36.9	32.2	18.0	3.6
**hsa-miR-22-3p**	27.2	27.4	27.0	27.0	26.8	26.8	26.5	26.9	28.0	34.2	33.6	32.1	33.2	31.9	29.9	34.3	34.9	31.4	18.0	3.2
**hsa-miR-365a-3p**	28.3	28.7	29.0	29.0	28.3	27.9	28.5	28.7	29.3	36.2	34.7	32.9	35.5	33.8	31.7	35.7	35.2	33.5	18.0	3.1
**hsa-let-7i-5p**	25.0	25.0	25.2	25.7	25.6	25.7	25.6	25.5	26.3	31.3	32.1	30.0	31.6	29.9	29.0	32.5	33.2	30.2	18.0	3.0
**hsa-miR-29c-3p**	27.0	27.5	27.2	28.6	28.1	27.4	28.9	29.0	30.5	33.8	33.6	31.9	33.2	31.7	30.5	35.0	36.5	32.5	18.0	2.9
**hsa-let-7b-5p**	25.6	25.8	24.8	25.0	25.4	25.7	24.5	24.7	25.3	30.7	31.9	29.7	31.9	28.9	28.2	31.8	32.3	28.8	18.0	2.9
**hsa-miR-193b-3p**	27.6	27.9	27.9	27.3	27.1	26.8	26.9	26.9	27.0	32.3	33.0	31.0	32.1	30.9	29.9	35.5	35.0	30.4	18.0	2.9
**hsa-let-7c**	26.3	26.9	26.3	26.5	27.0	27.2	26.1	26.2	27.4	32.1	32.1	30.7	32.2	30.5	29.3	33.2	33.6	30.0	18.0	2.7
**hsa-miR-127-3p**	27.6	28.0	27.8	27.8	27.6	27.5	27.6	27.5	27.7	33.1	35.0	31.6	32.4	30.7	29.8	33.6	34.0	30.7	18.0	2.7
**hsa-miR-31-5p**	24.9	25.1	24.9	26.3	25.9	26.0	28.0	27.9	28.8	29.6	30.6	28.9	30.8	29.0	28.8	31.6	34.4	31.0	18.0	2.6
**hsa-miR-214-3p**	27.0	27.1	26.9	26.6	26.6	26.9	26.5	26.2	26.9	31.7	32.6	31.3	32.5	30.8	29.3	32.6	33.1	30.0	18.0	2.6
**hsa-miR-29b-3p**	27.9	28.3	28.5	30.2	29.9	28.8	30.4	30.7	32.6	34.0	34.1	32.1	33.8	32.7	31.5	35.2	36.9	33.3	18.0	2.6
**hsa-miR-125a-5p**	26.2	26.2	26.2	26.2	26.0	26.0	25.6	25.8	25.9	31.3	32.2	30.2	31.1	29.6	28.5	31.2	32.2	30.0	18.0	2.6
**hsa-miR-199a-3p**	24.6	24.7	24.6	24.7	24.6	24.8	25.0	24.8	26.6	29.9	30.4	28.5	29.8	28.3	27.0	30.2	31.4	28.0	18.0	2.5
**hsa-let-7d-5p**	28.5	28.9	28.6	28.5	28.6	28.6	27.6	27.3	28.8	33.3	34.3	31.7	33.0	31.5	30.7	33.9	34.6	31.3	18.0	2.5
**hsa-miR-125b-5p**	22.3	22.5	22.7	23.2	22.8	22.8	23.1	23.3	23.8	27.2	27.8	26.6	27.8	26.0	25.6	28.2	29.9	26.8	18.0	2.4
**hsa-miR-31-3p**	28.4	28.6	28.8	29.8	29.2	29.1	32.3	31.9	33.0	32.8	32.7	31.6	32.5	31.3	31.0	35.8	36.8	34.2	18.0	2.4
**hsa-let-7f-5p**	28.6	28.6	28.5	28.2	28.3	28.4	27.6	27.1	30.7	32.9	33.3	32.3	32.9	31.6	31.1	34.1	34.0	31.2	18.0	2.4
**hsa-miR-423-3p**	28.0	28.0	28.2	28.1	28.2	28.3	28.1	27.9	28.6	33.0	32.7	31.8	32.6	32.1	30.5	33.8	34.2	31.8	18.0	2.4
**hsa-let-7g-5p**	26.9	27.0	26.9	26.8	27.0	27.1	26.9	26.6	28.1	32.2	32.3	30.0	31.3	30.0	29.0	33.1	32.7	29.8	18.0	2.4
**hsa-miR-107**	27.8	28.2	27.9	28.4	28.1	28.3	28.3	27.9	29.7	32.6	33.1	31.3	33.0	30.9	29.8	33.2	35.2	30.8	18.0	2.4
**hsa-miR-320a**	28.2	28.6	28.0	28.1	28.3	28.2	27.7	27.6	28.1	31.9	32.5	31.8	32.5	30.4	29.7	33.9	34.4	30.1	18.0	2.3
**hsa-miR-195-5p**	30.2	29.8	30.0	30.1	30.2	29.7	30.8	30.7	32.6	34.5	35.2	33.2	36.0	32.8	31.4	35.0	35.8	33.1	18.0	2.3
**hsa-miR-424-5p**	29.3	28.8	28.1	28.9	28.0	29.3	29.3	29.6	31.8	33.2	32.7	31.7	32.3	31.0	29.8	35.2	35.2	31.1	18.0	2.2
**hsa-miR-99b-5p**	28.5	28.6	28.6	28.7	28.6	28.8	28.3	28.5	28.9	33.1	33.8	32.3	33.0	31.1	29.9	33.9	33.7	31.3	18.0	2.2
**hsa-miR-103a-3p**	26.1	26.5	26.1	26.7	26.6	26.4	26.6	26.5	27.5	30.8	31.3	29.4	30.8	29.7	27.9	31.5	32.2	29.3	18.0	2.2
**hsa-miR-222-3p**	24.8	25.1	25.2	25.9	25.5	25.6	26.1	26.3	26.8	28.6	29.5	27.9	28.7	27.2	26.8	30.2	32.6	28.8	18.0	2.1
**hsa-miR-28-5p**	30.5	30.4	30.2	30.5	30.2	30.1	30.8	30.8	32.4	34.4	35.2	34.1	34.0	33.5	32.6	33.8	35.8	35.6	18.0	2.1
**hsa-miR-221-3p**	23.2	23.5	23.3	24.5	24.6	24.0	24.5	24.6	25.3	27.6	28.2	26.6	27.8	26.5	25.7	29.0	30.3	26.7	18.0	2.1
**hsa-miR-143-3p**	29.7	28.8	29.0	28.9	29.0	29.1	27.8	27.6	29.9	33.3	33.0	31.5	32.5	31.1	29.4	33.5	34.4	30.6	18.0	2.1

Supplementary data

**hsa-miR-15a-5p**	26.8	26.9	26.7	27.6	27.5	27.2	29.0	28.7	31.2	31.3	31.6	29.8	30.4	29.6	28.2	32.3	33.6	29.7	18.0	2.1
**hsa-miR-15b-5p**	28.7	29.3	28.6	29.2	29.5	29.0	29.2	29.3	30.5	32.2	32.8	31.3	32.3	30.8	29.9	35.8	34.6	31.1	18.0	2.1
**hsa-miR-574-3p**	29.1	29.1	28.8	29.1	28.8	28.7	28.9	28.7	28.9	32.9	32.8	31.8	32.8	31.7	30.9	33.3	34.9	32.3	18.0	2.1
**hsa-miR-19b-3p**	27.3	27.5	27.6	27.8	27.5	26.8	29.0	29.2	31.0	31.6	31.0	29.1	29.7	29.1	28.2	32.3	34.6	29.9	18.0	2.1
**hsa-miR-379-5p**	29.7	29.8	29.7	29.9	29.8	30.0	30.6	30.2	31.9	34.9	33.4	32.2	33.8	32.2	32.1	34.2	36.2	32.9	18.0	2.0
**hsa-miR-30a-5p**	31.5	31.5	31.2	31.8	31.5	30.8	30.1	30.1	31.5	35.6	34.8	34.1	34.5	32.7	31.2	34.2	36.8	31.2	18.0	2.0
**hsa-miR-19a-3p**	28.1	28.6	28.7	29.0	28.3	27.6	29.8	30.4	31.8	31.6	31.2	29.6	30.7	30.0	28.8	32.8	35.8	30.8	18.0	2.0
**hsa-miR-24-3p**	25.1	25.0	25.0	25.5	25.4	25.2	25.0	24.9	26.3	29.0	29.7	28.1	29.2	27.8	26.8	29.5	30.8	27.3	18.0	2.0
**hsa-miR-181a-5p**	28.0	28.3	28.2	28.5	28.3	28.7	27.1	27.2	27.6	32.3	32.7	30.3	31.5	30.3	28.8	31.9	33.3	29.8	18.0	2.0
**hsa-miR-132-3p**	30.5	30.3	31.0	30.2	29.8	30.5	30.0	30.5	30.4	34.3	33.2	33.9	35.3	32.1	31.1	33.7	36.0	31.6	18.0	2.0
**hsa-miR-148b-3p**	30.2	30.0	30.4	30.8	30.5	30.2	30.8	30.7	31.8	33.9	34.5	32.1	33.1	32.5	31.8	35.1	36.8	33.3	18.0	2.0
**hsa-miR-652-3p**	32.5	32.3	31.8	32.5	31.9	32.0	31.6	31.9	31.9	36.9	35.1	33.1	36.1	33.8	31.7	36.3	37.0	32.8	18.0	1.9
**hsa-miR-374b-5p**	31.3	32.0	31.5	31.8	31.2	30.9	31.8	32.1	34.1	34.5	35.5	33.6	36.5	33.4	33.0	36.2	36.5	32.8	18.0	1.9
**hsa-miR-152**	27.8	27.9	27.7	27.8	27.7	27.8	28.0	28.0	29.7	32.1	31.8	30.8	31.6	30.6	29.4	31.7	33.1	30.0	18.0	1.9
**hsa-miR-382-5p**	30.3	30.9	30.1	30.7	31.1	31.0	31.0	30.6	31.3	33.2	35.7	33.2	34.3	32.8	31.3	35.0	35.7	32.5	18.0	1.9
**hsa-miR-423-5p**	29.2	29.7	29.2	29.2	29.6	29.6	29.2	29.1	29.2	32.2	32.3	31.7	31.9	31.6	30.1	33.8	35.3	31.2	18.0	1.8
**hsa-miR-26b-5p**	30.4	31.0	29.9	30.7	30.7	30.8	30.6	30.9	32.8	34.6	34.8	33.2	34.6	32.0	31.0	35.1	34.7	32.4	18.0	1.8
**hsa-miR-191-5p**	28.6	29.0	28.5	29.0	29.0	28.8	29.0	29.1	29.7	32.7	32.9	31.6	32.7	31.3	30.2	32.9	33.7	31.3	18.0	1.8
**hsa-miR-99a-5p**	28.5	29.5	28.6	28.9	28.8	29.1	29.1	29.0	30.7	32.3	32.9	31.8	32.5	30.6	29.7	33.3	33.9	30.9	18.0	1.8
**hsa-miR-23b-3p**	27.0	26.9	26.9	27.4	27.2	27.0	26.5	26.7	27.8	30.8	31.0	29.5	30.7	29.1	27.8	31.0	31.7	28.8	18.0	1.8
**hsa-miR-140-3p**	30.5	30.9	30.2	31.0	31.1	30.2	30.7	30.4	31.1	35.0	32.9	32.5	33.3	32.0	31.3	33.5	36.9	32.5	18.0	1.8
**hsa-miR-361-5p**	28.4	28.8	28.6	28.8	28.6	28.5	29.0	28.7	30.0	32.5	33.5	31.1	32.0	31.1	30.0	32.1	33.5	30.5	18.0	1.8
**hsa-miR-23a-3p**	24.7	24.7	24.8	25.4	25.0	24.7	24.7	24.9	26.0	28.4	28.6	27.2	28.4	26.9	25.8	28.8	30.2	26.9	18.0	1.8
**hsa-miR-151a-5p**	28.2	28.2	28.3	28.6	28.5	28.6	28.8	28.8	29.9	31.8	32.5	30.7	32.5	30.7	29.7	32.4	33.5	30.9	18.0	1.8
**hsa-miR-21-5p**	22.7	23.0	23.0	23.2	22.9	23.0	22.9	23.0	24.7	26.3	26.9	25.6	26.6	25.1	23.9	27.0	27.8	24.7	18.0	1.7
**hsa-miR-30c-5p**	28.8	29.5	29.2	29.8	29.0	28.7	29.6	29.6	30.9	32.9	32.3	30.8	32.2	30.7	29.9	32.7	34.1	30.8	18.0	1.6
**hsa-miR-16-5p**	24.1	24.3	24.3	25.0	24.9	24.8	25.9	25.7	27.2	27.6	28.4	26.5	27.5	26.5	25.5	28.2	29.6	26.9	18.0	1.6
**hsa-miR-151a-3p**	31.4	31.8	31.4	31.9	31.7	32.2	33.0	32.4	35.3	34.6	34.6	34.0	34.3	33.5	32.7	36.4	36.1	34.0	18.0	1.6
**hsa-miR-301a-3p**	30.7	31.2	30.9	33.3	31.9	31.9	33.6	32.9	35.7	33.1	33.5	31.7	32.6	32.0	31.1	33.2	36.6	31.7	18.0	1.6
**hsa-miR-376a-3p**	32.1	31.4	32.0	31.2	32.0	31.0	32.4	31.9	35.0	34.2	34.5	33.7	34.1	33.2	32.3	35.9	36.1	33.5	18.0	1.6
**hsa-miR-411-5p**	29.8	30.3	29.9	30.7	30.6	30.1	31.3	32.4	33.0	33.4	34.3	32.1	32.6	32.2	31.1	34.5	34.4	31.8	18.0	1.6
**hsa-miR-10b-5p**	28.8	29.1	28.9	27.7	28.2	28.2	28.7	29.2	30.6	32.1	32.6	30.6	31.6	30.2	29.5	31.8	32.1	29.5	18.0	1.5
**hsa-miR-30e-5p**	29.8	30.2	29.8	30.4	30.6	29.6	31.7	31.7	32.8	33.8	33.2	31.7	32.7	31.3	30.5	33.6	34.8	31.8	18.0	1.5

Supplementary data

**hsa-miR-154-5p**	30.9	31.8	30.9	31.6	30.7	30.5	31.2	31.7	32.1	34.5	34.1	33.6	34.8	33.9	31.5	33.9	34.9	32.6	18.0	1.5
**hsa-miR-186-5p**	29.8	30.6	30.1	30.5	29.6	29.3	30.2	30.5	31.4	33.0	32.9	31.0	32.8	31.1	30.0	33.4	34.7	31.2	18.0	1.5
**hsa-miR-27b-3p**	27.7	27.6	27.3	28.1	27.1	27.3	27.2	27.0	28.3	30.7	30.7	29.5	30.4	28.9	27.8	30.4	31.6	28.3	18.0	1.5
**hsa-miR-30b-5p**	30.1	30.1	29.3	30.2	29.7	29.2	30.0	30.0	31.7	33.0	32.7	31.0	32.7	31.0	29.8	32.9	34.3	31.6	18.0	1.5
**hsa-miR-106a-5p**	27.7	27.8	28.0	28.0	28.1	27.6	30.0	29.5	31.5	30.5	31.0	29.3	30.5	29.6	28.5	31.8	31.9	29.5	18.0	1.5
**hsa-miR-20a-5p**	27.2	27.5	27.7	27.7	27.6	27.4	29.6	29.4	31.3	30.0	30.7	29.2	30.0	28.9	27.9	30.8	31.7	29.3	18.0	1.5
**hsa-miR-93-5p**	28.5	28.3	28.5	29.3	29.1	28.8	29.8	29.7	30.6	31.0	31.8	29.6	30.7	29.7	28.7	31.6	33.8	30.0	18.0	1.4
**hsa-miR-101-3p**	31.4	31.4	30.9	31.3	30.3	30.6	30.9	31.1	33.5	34.1	33.5	31.9	32.7	31.7	30.1	33.1	34.8	32.2	18.0	1.4
**hsa-miR-18b-5p**	30.9	30.7	31.1	32.1	31.3	30.8	34.5	32.8	33.6	33.5	33.6	32.1	33.9	32.7	30.9	33.6	34.7	31.8	18.0	1.4
**hsa-miR-92a-3p**	27.4	27.6	27.5	27.5	27.5	27.1	27.9	27.8	28.2	29.8	30.3	29.0	29.3	28.6	27.6	30.2	32.0	28.8	18.0	1.3
**hsa-miR-18a-5p**	31.5	30.7	31.9	31.9	31.9	30.9	33.5	33.7	34.5	34.6	33.9	32.2	33.0	32.0	31.3	33.7	33.9	32.3	18.0	1.2
**hsa-miR-425-5p**	31.7	31.6	32.3	32.3	31.8	31.8	32.0	32.6	32.6	34.6	35.2	32.9	33.5	32.8	31.9	34.6	35.2	33.0	18.0	1.2
**hsa-let-7d-3p**	32.2	31.0	31.1	32.0	31.6	31.4	31.9	31.9	31.7	32.7	33.3	31.6	32.3	31.9	30.5	33.2	35.9	31.5	18.0	1.2
**hsa-miR-25-3p**	30.1	30.0	29.9	30.8	30.8	30.3	31.6	31.4	32.5	32.4	32.6	30.6	31.1	30.8	29.7	32.2	33.7	30.9	18.0	1.1
**hsa-miR-126-3p**	33.1	34.5	34.5	34.8	36.2	34.6	35.7	37.0	ND	30.8	29.4	27.8	28.3	27.2	25.9	28.9	29.7	26.7	17.0	3.7
**hsa-miR-29a-3p**	25.0	25.5	25.5	26.8	26.3	25.6	ND	27.3	28.4	32.0	31.7	30.0	30.9	29.7	28.8	32.8	35.6	31.1	17.0	3.1
**hsa-miR-199a-5p**	26.5	26.5	26.2	26.0	25.8	25.6	26.8	26.6	27.6	33.1	33.7	31.1	32.6	31.0	29.3	33.1	BF	30.8	17.0	3.0
**hsa-let-7e-5p**	26.8	27.1	26.9	27.0	27.0	26.9	26.2	26.1	26.8	32.0	33.5	31.3	ND	30.6	30.0	32.2	33.4	30.1	17.0	2.7
**hsa-miR-196a-5p**	30.3	31.0	30.6	30.1	30.5	30.6	31.0	29.7	31.7	36.5	36.6	33.9	BF	34.2	32.8	36.5	36.9	34.3	17.0	2.6
**hsa-let-7a-5p**	23.1	23.1	22.8	23.1	ND	23.3	22.8	22.6	23.8	27.9	29.0	27.1	28.5	26.8	25.9	28.9	29.8	26.6	17.0	2.6
**hsa-miR-145-5p**	28.0	27.6	27.6	27.6	ND	27.1	24.9	24.8	25.6	31.3	30.9	29.6	30.8	29.2	27.8	31.0	32.3	28.0	17.0	2.3
**hsa-miR-181b-5p**	31.6	31.6	31.1	32.1	32.8	32.1	30.2	30.3	31.0	36.7	35.8	35.2	35.9	34.3	33.4	36.7	ND	34.1	17.0	2.2
**hsa-miR-199b-5p**	26.5	26.9	26.9	27.3	27.0	27.0	27.7	27.7	29.5	31.6	32.8	30.5	32.0	30.5	29.0	32.5	BF	30.6	17.0	2.2
**hsa-miR-486-5p**	34.7	34.1	ND	35.2	34.0	34.5	35.8	35.2	36.6	31.8	32.3	30.4	31.1	30.9	29.9	32.5	33.5	30.6	17.0	2.1
**hsa-miR-708-5p**	29.1	28.9	29.8	31.0	30.5	30.4	31.2	31.7	32.3	32.7	33.8	32.3	35.6	32.2	32.3	35.6	ND	34.8	17.0	2.1
**hsa-miR-100-5p**	24.2	24.5	24.5	25.5	25.2	25.1	25.8	25.9	26.8	28.2	29.0	27.6	ND	27.1	26.6	29.3	30.7	27.7	17.0	1.9
**hsa-miR-26a-5p**	25.9	26.2	25.7	26.3	26.0	26.0	26.0	26.0	27.5	30.3	30.5	28.9	30.1	28.8	27.5	30.7	BF	28.2	17.0	1.9
**hsa-miR-155-5p**	30.0	30.1	30.1	31.2	30.9	31.0	33.2	32.6	32.9	33.9	35.9	33.1	BF	33.4	32.3	34.7	35.4	34.2	17.0	1.8
**hsa-miR-374a-5p**	31.1	31.6	31.4	32.0	31.5	30.7	31.8	32.0	34.6	34.7	35.8	34.2	35.7	33.6	33.2	35.7	BF	35.1	17.0	1.8
**hsa-miR-98-5p**	32.0	31.9	32.1	32.2	32.2	32.6	32.2	31.7	34.1	36.6	35.7	34.2	36.2	34.7	34.5	BF	36.6	34.9	17.0	1.8
**hsa-miR-487b**	31.6	32.2	32.3	32.7	31.8	31.5	32.0	32.3	32.8	36.3	35.5	35.0	35.6	34.9	32.9	36.3	BF	34.8	17.0	1.7
**hsa-miR-484**	30.0	30.3	30.8	30.8	30.5	30.3	30.7	30.5	30.8	33.8	34.0	32.8	34.0	32.9	32.0	35.5	BF	33.7	17.0	1.7
**hsa-miR-409-3p**	28.8	29.1	29.6	29.5	29.1	29.2	29.6	29.2	29.7	32.5	33.9	31.5	ND	32.2	30.6	33.2	33.2	31.5	17.0	1.7

Supplementary data

**hsa-miR-337-3p**	31.3	31.8	31.9	31.8	30.9	30.9	32.0	32.0	35.2	BF	34.5	35.1	34.5	33.5	33.0	34.7	36.6	32.8	17.0	1.7
**hsa-miR-106b-5p**	28.6	29.2	29.2	30.0	29.7	28.9	31.4	30.8	32.9	32.5	33.6	30.7	32.2	30.7	29.9	33.9	BF	31.2	17.0	1.6
**hsa-miR-376b-3p**	32.1	32.1	32.6	33.0	32.0	31.9	33.8	33.9	35.1	36.6	35.9	34.7	35.2	34.2	33.2	36.5	ND	34.7	17.0	1.6
**hsa-miR-140-5p**	30.8	31.0	31.2	31.7	31.0	30.8	31.9	31.5	32.6	34.9	34.5	33.6	34.9	32.6	32.8	34.3	ND	32.7	17.0	1.5
**hsa-miR-532-5p**	32.2	32.1	32.1	33.0	32.1	32.1	31.5	32.7	32.8	35.3	35.3	34.8	34.1	34.8	33.8	35.1	BF	34.2	17.0	1.3
**hsa-miR-323a-3p**	33.0	32.5	33.1	33.9	33.5	33.2	33.7	34.5	34.7	36.1	35.2	34.2	35.3	35.9	34.6	BF	36.7	36.7	17.0	1.3
**hsa-miR-660-5p**	31.9	32.1	31.6	33.0	31.4	31.2	31.9	32.6	33.1	34.4	34.1	34.2	34.1	33.6	32.0	34.7	ND	33.7	17.0	1.2
**hsa-miR-378a-3p**	32.6	32.3	32.2	32.5	33.1	32.4	34.6	34.9	34.4	34.9	34.3	33.7	35.0	33.5	32.3	35.6	ND	34.4	17.0	1.1
**hsa-miR-215**	35.8	36.8	BF	36.9	35.6	36.7	35.8	36.9	36.8	36.9	36.9	35.5	36.4	34.8	33.7	37.0	35.5	34.5	17.0	1.0
**hsa-miR-194-5p**	36.2	34.8	34.7	35.0	36.3	35.1	BF	35.5	36.7	35.7	36.1	34.2	34.9	34.6	33.1	36.7	36.3	34.7	17.0	1.0
**hsa-miR-130a-3p**	29.6	30.0	29.6	29.8	29.8	29.3	30.1	30.1	31.6	36.8	BF	34.5	34.9	34.1	32.3	ND	36.5	34.0	16.0	2.7
**hsa-miR-27a-3p**	28.1	28.9	ND	28.1	28.2	27.7	28.0	28.1	30.7	ND	31.6	31.2	32.4	30.7	28.8	33.9	34.0	30.1	16.0	2.1
**hsa-miR-342-3p**	30.2	30.7	30.2	30.8	30.6	30.7	30.5	30.7	31.2	ND	35.8	32.7	34.5	33.2	31.8	35.2	ND	32.8	16.0	1.8
**hsa-miR-432-5p**	32.1	32.8	32.1	32.8	32.7	32.6	32.2	31.8	32.1	36.2	BF	35.8	35.9	35.9	33.2	BF	36.5	33.9	16.0	1.7
**hsa-miR-454-3p**	31.6	31.7	30.8	32.0	32.4	32.6	32.2	32.7	34.1	35.3	36.0	34.8	35.1	34.9	33.0	BF	ND	34.1	16.0	1.6
**hsa-miR-493-3p**	32.1	32.5	32.5	32.3	33.1	32.5	32.8	32.7	33.6	36.8	ND	34.8	35.7	35.0	33.9	ND	36.6	34.6	16.0	1.6
**hsa-miR-505-3p**	33.1	33.0	33.2	33.6	32.7	32.8	32.2	32.9	33.2	BF	35.7	35.6	35.2	34.0	33.0	36.7	ND	34.1	16.0	1.3
**hsa-miR-590-5p**	33.4	33.5	34.1	35.0	33.8	33.4	35.5	34.7	36.6	BF	36.8	35.6	36.1	36.1	34.7	BF	36.5	35.5	16.0	1.2
**hsa-miR-17-5p**	33.7	33.2	34.0	33.4	34.5	34.2	36.3	34.7	35.2	34.9	36.2	35.9	35.7	35.1	33.3	BF	ND	35.5	16.0	1.0
**hsa-miR-410**	33.6	34.6	33.8	34.5	33.7	33.7	33.6	35.2	34.8	35.9	35.7	34.7	35.0	34.3	33.4	BF	ND	34.9	16.0	0.8
**hsa-miR-497-5p**	30.2	30.1	30.2	30.5	30.1	30.0	30.9	30.7	31.9	ND	BF	35.5	34.7	34.5	33.0	36.7	ND	33.6	15.0	2.3
**hsa-miR-193a-5p**	29.7	30.1	29.8	30.6	30.1	30.3	ND	30.7	30.6	35.0	34.6	34.4	35.5	33.2	32.8	BF	BF	35.1	15.0	2.3
**hsa-miR-22-5p**	30.2	30.0	30.1	30.1	30.1	29.8	30.8	29.9	31.8	ND	ND	35.2	35.4	34.1	32.8	34.8	ND	34.2	15.0	2.2
**hsa-miR-324-5p**	31.6	31.7	31.3	32.5	31.4	31.3	31.8	31.6	32.1	35.9	BF	34.2	36.8	BF	34.9	36.3	BF	35.8	15.0	2.1
**hsa-miR-92b-3p**	31.8	32.2	31.5	31.0	31.9	31.1	31.1	31.5	31.5	35.6	36.9	34.7	BF	34.1	33.8	BF	ND	34.5	15.0	1.9
**hsa-miR-299-5p**	31.1	31.7	31.8	31.7	31.5	30.9	31.7	32.0	32.8	BF	35.5	35.1	35.9	35.1	33.4	35.8	ND	ND	15.0	1.9
**hsa-miR-137**	31.5	31.4	31.8	32.2	31.9	32.8	31.5	31.6	34.3	35.9	36.3	35.8	BF	35.2	34.5	BF	BF	34.5	15.0	1.9
**hsa-miR-328**	32.9	33.4	32.8	33.1	31.5	32.7	33.1	32.9	33.2	ND	BF	36.8	36.9	36.6	34.3	36.5	ND	36.2	15.0	1.8
**hsa-miR-218-5p**	30.7	31.0	31.1	32.3	31.6	31.4	33.6	33.4	35.9	34.7	35.4	35.1	35.2	34.2	34.0	BF	ND	BF	15.0	1.8
**hsa-miR-30e-3p**	31.9	32.5	32.1	32.7	32.1	32.8	30.9	31.7	33.2	35.6	BF	36.0	36.8	35.3	32.3	BF	BF	33.5	15.0	1.8
**hsa-miR-149-5p**	32.7	33.0	31.9	32.9	34.1	31.8	32.4	33.1	31.9	BF	36.3	36.0	BF	36.7	33.7	36.3	ND	34.8	15.0	1.8
**hsa-miR-485-3p**	32.2	32.9	32.7	33.0	32.8	31.8	31.9	31.7	32.4	36.2	35.5	ND	35.7	35.0	35.2	ND	ND	35.3	15.0	1.6
**hsa-miR-210**	31.8	32.6	32.7	34.2	33.0	32.1	33.6	ND	33.6	35.6	35.6	34.7	35.8	BF	33.5	BF	36.9	34.8	15.0	1.5

Supplementary data

**hsa-miR-224-5p**	32.8	32.4	32.6	33.0	33.1	33.2	35.2	34.5	35.6	BF	36.0	35.2	36.8	35.3	34.8	BF	ND	35.3	15.0	1.4
**hsa-miR-185-5p**	33.0	33.3	33.0	33.2	ND	34.0	33.9	33.7	35.9	36.2	ND	34.8	35.5	34.4	33.0	36.9	BF	34.2	15.0	1.3
**hsa-miR-204-5p**	33.0	BF	33.0	34.2	34.8	32.0	34.9	36.1	34.0	36.2	ND	33.9	35.5	34.2	33.2	35.1	ND	33.7	15.0	1.2
**hsa-miR-192-5p**	35.8	36.2	35.5	33.9	35.1	34.2	ND	36.1	ND	34.1	34.7	33.6	34.3	35.0	32.2	36.3	ND	33.2	15.0	1.2
**hsa-miR-503-5p**	32.6	30.6	31.2	31.4	30.3	31.2	33.0	32.2	34.5	36.5	35.3	36.5	ND	36.9	34.9	ND	ND	ND	14.0	2.4
**hsa-miR-136-5p**	28.7	28.9	28.8	29.4	28.5	28.2	29.7	29.9	31.1	ND	BF	34.1	34.6	33.9	31.9	BF	BF	33.5	14.0	2.4
**hsa-miR-197-3p**	28.9	29.5	29.2	29.9	29.4	29.7	ND	29.5	29.2	BF	33.7	33.5	ND	33.0	32.3	ND	36.0	32.2	14.0	2.3
**hsa-miR-130b-3p**	31.9	32.2	32.3	33.4	33.5	32.8	33.2	33.5	35.5	BF	BF	35.8	35.9	34.3	34.2	ND	ND	35.7	14.0	1.4
**hsa-miR-425-3p**	33.7	34.4	34.6	34.2	33.5	33.6	33.7	33.5	34.2	ND	37.0	BF	36.9	35.8	33.7	ND	BF	36.8	14.0	1.3
**hsa-miR-539-5p**	32.3	33.0	32.7	32.8	33.5	32.4	33.6	32.8	35.1	36.0	ND	36.0	ND	35.1	34.7	ND	ND	34.5	14.0	1.3
**hsa-miR-146b-5p**	33.2	33.2	32.8	35.0	34.3	33.0	34.0	33.6	36.0	36.8	BF	34.6	ND	36.6	35.0	ND	ND	35.0	14.0	1.3
**hsa-miR-455-5p**	33.2	32.8	33.4	34.3	33.0	32.7	32.3	33.7	34.4	35.9	35.0	35.8	ND	35.5	34.8	ND	ND	BF	14.0	1.2
**hsa-miR-30d-5p**	33.7	34.8	33.8	34.8	34.1	33.4	34.1	33.7	35.8	BF	ND	36.2	36.8	36.6	33.9	BF	ND	35.4	14.0	1.2
**hsa-miR-452-5p**	34.5	34.8	33.4	34.9	34.8	34.2	36.9	34.8	36.7	ND	BF	35.4	ND	36.1	36.3	ND	36.9	36.5	14.0	1.1
**hsa-miR-301b**	33.8	35.0	33.9	34.8	33.9	35.5	36.9	36.3	BF	ND	37.0	35.1	35.6	36.7	35.5	BF	ND	34.5	14.0	1.1
**hsa-miR-377-3p**	30.9	32.0	31.5	32.1	30.9	31.0	32.6	32.8	34.6	BF	BF	36.4	36.7	BF	34.8	ND	BF	35.0	13.0	2.1
**hsa-miR-196b-5p**	31.7	31.6	32.0	32.2	31.9	32.1	31.9	31.5	32.7	ND	ND	35.7	ND	34.8	BF	36.7	ND	36.3	13.0	1.9
**hsa-miR-337-5p**	32.6	33.2	32.3	32.6	32.7	33.0	34.0	33.2	34.8	ND	BF	36.5	BF	36.3	35.0	ND	ND	36.8	13.0	1.6
**hsa-miR-339-5p**	32.5	32.6	33.5	33.4	33.4	33.1	33.6	35.1	34.6	ND	BF	36.3	BF	36.7	36.6	36.9	ND	ND	13.0	1.6
**hsa-miR-629-5p**	33.6	33.0	32.8	34.3	35.3	35.1	34.8	35.7	35.1	36.1	ND	36.4	ND	37.0	35.7	ND	ND	ND	13.0	1.3
**hsa-miR-433**	34.0	33.5	34.0	33.9	33.9	33.9	34.6	33.9	33.6	BF	BF	36.6	ND	36.6	34.0	ND	BF	34.9	13.0	1.0
**hsa-miR-10a-5p**	33.3	33.1	33.0	33.0	34.1	33.5	34.0	33.2	34.8	35.3	BF	BF	BF	35.0	34.7	BF	ND	36.1	13.0	1.0
**hsa-miR-421**	33.9	33.9	33.5	34.7	34.0	33.9	34.1	35.9	34.5	ND	ND	34.2	37.0	BF	34.3	ND	ND	35.4	13.0	1.0
**hsa-miR-877-5p**	34.5	36.1	ND	34.6	35.1	34.5	35.0	34.9	34.1	35.9	BF	34.8	ND	34.2	33.9	35.4	ND	ND	13.0	0.7
**hsa-miR-138-5p**	30.5	ND	31.1	ND	33.2	32.5	33.6	33.5	ND	36.2	36.9	ND	36.8	34.8	34.3	ND	ND	36.9	12.0	2.2
**hsa-miR-95**	36.2	ND	ND	35.9	35.2	33.8	31.6	31.3	33.2	35.0	ND	35.6	ND	35.5	35.8	ND	ND	33.6	12.0	1.7
**hsa-miR-874**	32.4	33.1	33.0	33.6	32.9	33.6	31.7	32.4	32.6	ND	ND	ND	35.1	36.7	34.5	ND	ND	BF	12.0	1.4
**hsa-miR-331-3p**	34.4	33.1	34.6	33.2	33.6	34.1	32.3	33.5	34.0	ND	BF	36.1	BF	36.0	35.0	BF	ND	BF	12.0	1.1
**hsa-miR-766-3p**	34.7	35.8	34.1	ND	35.2	35.5	32.6	ND	35.0	36.0	ND	36.0	ND	36.1	ND	36.1	ND	36.1	12.0	1.1
**hsa-miR-99a-3p**	33.1	33.6	33.8	34.3	33.1	33.6	35.0	33.9	ND	ND	ND	36.1	ND	34.8	35.9	ND	ND	35.2	12.0	1.0
**hsa-miR-212-3p**	34.2	34.6	35.3	34.3	33.1	36.0	33.9	33.9	33.8	ND	ND	ND	ND	36.0	34.5	ND	ND	35.0	12.0	0.9
**hsa-miR-335-5p**	36.6	35.2	36.9	35.9	36.7	34.8	BF	ND	BF	BF	35.7	36.0	35.7	35.5	34.2	ND	ND	34.9	12.0	0.8
**hsa-miR-32-5p**	32.1	32.8	32.1	32.3	31.6	31.2	32.7	32.9	34.9	ND	ND	ND	ND	37.0	35.9	ND	ND	ND	11.0	1.9

Supplementary data

**hsa-miR-370**	30.8	30.7	31.6	31.9	30.9	30.2	31.3	31.1	31.7	ND	ND	ND	BF	BF	35.8	BF	ND	34.9	11.0	1.8
**hsa-miR-33a-5p**	31.8	32.2	32.4	32.9	32.0	31.6	32.9	32.5	35.0	ND	ND	ND	36.9	BF	35.3	ND	ND	ND	11.0	1.7
**hsa-miR-34c-5p**	32.6	32.1	31.8	32.6	31.6	31.7	32.5	32.5	33.3	ND	ND	ND	ND	36.1	BF	BF	ND	35.9	11.0	1.6
**hsa-miR-615-3p**	31.8	32.5	32.5	32.2	32.7	32.2	31.9	31.9	32.0	BF	BF	36.3	ND	ND	35.4	ND	ND	BF	11.0	1.5
**hsa-miR-146a-5p**	33.9	33.3	34.0	36.5	34.2	35.4	36.6	36.1	BF	ND	ND	BF	ND	37.0	36.8	ND	ND	35.9	11.0	1.3
**hsa-miR-542-5p**	32.9	33.8	33.6	33.8	32.7	35.1	35.1	33.3	34.9	ND	ND	ND	ND	36.1	36.1	ND	ND	ND	11.0	1.2
**hsa-miR-7-5p**	33.5	32.9	34.0	34.2	33.8	33.0	34.5	35.0	BF	36.3	ND	35.5	ND	BF	36.0	ND	ND	BF	11.0	1.2
**hsa-miR-361-3p**	32.8	33.5	33.1	34.7	33.2	33.9	34.1	33.9	35.1	ND	BF	BF	36.7	ND	35.3	ND	ND	ND	11.0	1.1
**hsa-miR-21-3p**	32.3	32.5	33.5	33.8	32.5	33.1	32.9	33.1	34.0	BF	ND	BF	ND	35.4	35.5	BF	ND	BF	11.0	1.1
**hsa-miR-501-5p**	35.3	35.8	34.2	34.8	34.7	34.9	35.9	33.4	34.9	ND	ND	ND	ND	ND	36.0	ND	ND	36.7	11.0	0.9
**hsa-miR-133a**	BF	BF	ND	ND	ND	BF	34.7	36.8	37.0	35.8	36.8	35.9	35.3	35.5	34.7	36.3	ND	35.2	11.0	0.8
**hsa-miR-18a-3p**	34.5	34.4	35.7	35.9	34.3	35.1	ND	BF	35.3	36.2	ND	36.3	ND	ND	34.5	BF	ND	36.1	11.0	0.8
**hsa-miR-451a**	ND	36.7	ND	ND	ND	ND	ND	ND	ND	29.8	29.9	27.3	28.1	27.8	26.1	30.0	31.5	27.9	10.0	3.0
**hsa-miR-142-3p**	ND	BF	35.8	BF	ND	ND	ND	ND	ND	29.3	29.1	27.2	28.3	27.5	26.6	29.1	30.4	27.2	10.0	2.6
**hsa-miR-223-3p**	ND	ND	ND	ND	35.5	ND	ND	ND	ND	29.9	29.6	28.0	29.1	28.2	26.8	29.7	30.5	28.6	10.0	2.3
**hsa-miR-139-5p**	ND	BF	ND	ND	ND	36.8	ND	ND	ND	35.3	34.9	31.9	33.8	32.5	31.4	33.9	34.8	31.5	10.0	1.8
**hsa-miR-381-3p**	32.3	32.9	32.4	33.4	32.1	32.6	34.1	35.1	BF	ND	ND	35.3	BF	BF	35.3	ND	ND	BF	10.0	1.3
**hsa-miR-154-3p**	33.6	33.9	34.6	35.3	34.0	34.1	35.7	35.8	36.8	ND	ND	BF	ND	BF	36.8	ND	ND	ND	10.0	1.2
**hsa-miR-190a**	34.1	34.0	33.7	33.5	33.1	33.7	34.1	34.1	35.9	ND	ND	ND	ND	36.8	BF	ND	ND	ND	10.0	1.2
**hsa-miR-491-5p**	32.9	32.7	33.5	34.1	33.2	34.5	35.1	33.2	34.1	BF	ND	ND	ND	BF	ND	ND	ND	36.4	10.0	1.1
**hsa-miR-330-3p**	36.8	34.6	35.0	36.8	34.1	35.5	33.9	35.9	35.1	ND	ND	ND	ND	ND	35.5	ND	ND	ND	10.0	1.0
**hsa-miR-500a-5p**	36.7	36.4	36.4	35.5	36.1	35.8	33.5	34.8	35.4	BF	ND	BF	BF	ND	35.2	ND	ND	BF	10.0	1.0
**hsa-miR-744-5p**	33.2	33.7	33.4	34.1	34.2	33.9	33.8	33.7	33.9	BF	ND	BF	ND	BF	36.2	ND	ND	BF	10.0	0.8
**hsa-miR-654-5p**	34.0	34.8	35.6	34.7	36.0	35.0	35.0	34.6	34.0	ND	ND	ND	35.7	ND	ND	ND	ND	ND	10.0	0.7
**hsa-miR-376c-3p**	29.3	29.5	29.6	29.6	29.2	29.6	30.5	30.3	BF	BF	BF	BF	BF	31.3	30.6	BF	BF	BF	10.0	0.7
**hsa-miR-188-5p**	35.9	35.2	35.5	35.9	36.9	36.0	35.9	35.6	36.0	ND	ND	37.0	ND	ND	ND	ND	ND	ND	10.0	0.6
**hsa-miR-148a-3p**	30.1	30.9	30.4	30.9	30.3	30.1	31.2	31.0	BF	BF	BF	BF	BF	BF	31.2	BF	BF	31.9	10.0	0.6
**hsa-miR-122-5p**	ND	ND	ND	ND	BF	ND	ND	ND	ND	32.3	32.6	31.3	31.6	31.3	29.8	32.7	36.6	31.1	9.0	1.9
**hsa-miR-144-3p**	ND	ND	ND	ND	ND	ND	ND	ND	ND	33.9	33.3	31.4	32.1	31.7	30.1	34.8	36.0	32.0	9.0	1.9
**hsa-miR-150-5p**	BF	ND	ND	ND	ND	ND	ND	ND	ND	32.7	32.1	29.8	31.8	30.3	29.2	32.3	32.7	30.2	9.0	1.4
**hsa-miR-193a-3p**	33.7	34.3	34.2	34.3	34.2	33.2	35.6	36.3	36.6	ND	ND	ND	ND	ND	BF	ND	ND	BF	9.0	1.2
**hsa-miR-671-5p**	34.9	33.8	34.5	35.2	33.7	34.6	35.7	35.3	36.6	ND	ND	BF	ND	ND	ND	ND	ND	ND	9.0	0.9
**hsa-miR-296-5p**	32.7	32.8	33.2	32.3	32.2	32.9	32.5	32.5	31.8	ND	ND	ND	ND	ND	ND	ND	ND	ND	9.0	0.4

Supplementary data

**hsa-miR-324-3p**	30.3	31.1	31.2	31.2	30.7	30.6	30.7	30.3	30.8	BF	BF	BF	ND	BF	BF	BF	BF	BF	9.0	0.4
**hsa-miR-29b-2-5p**	ND	BF	ND	36.7	34.7	34.6	33.9	34.5	35.1	ND	ND	ND	ND	36.1	ND	36.3	BF	ND	8.0	1.0
**hsa-miR-127-5p**	34.8	37.0	34.9	36.1	34.0	35.0	36.4	35.5	BF	ND	ND	BF	ND	ND	ND	ND	ND	ND	8.0	1.0
**hsa-miR-584-5p**	35.7	34.9	34.7	BF	34.9	34.9	36.9	ND	36.7	BF	ND	ND	36.2	BF	ND	ND	ND	ND	8.0	0.9
**hsa-miR-133b**	BF	ND	36.6	ND	ND	ND	36.7	35.1	BF	ND	36.5	BF	35.5	35.7	35.1	36.1	ND	BF	8.0	0.6
**hsa-miR-326**	35.0	ND	36.6	35.9	36.6	36.6	36.4	ND	36.6	ND	BF	BF	ND	ND	37.0	BF	ND	ND	8.0	0.6
**hsa-miR-181d**	35.8	36.1	36.1	ND	35.5	36.7	35.5	BF	34.7	ND	ND	ND	ND	ND	35.7	ND	ND	BF	8.0	0.6
**hsa-miR-450a-5p**	36.5	36.2	36.9	36.6	35.8	35.6	BF	BF	BF	ND	BF	36.9	BF	BF	36.9	ND	ND	ND	8.0	0.5
**hsa-miR-494**	36.7	36.7	36.3	36.4	35.7	BF	36.2	BF	BF	BF	ND	BF	BF	35.9	35.9	BF	ND	ND	8.0	0.4
**hsa-miR-431-5p**	ND	ND	ND	ND	ND	32.8	ND	32.2	ND	BF	ND	BF	36.5	36.6	35.1	36.3	ND	35.9	7.0	1.8
**hsa-miR-135b-5p**	34.2	33.5	35.3	35.2	35.4	36.3	ND	ND	ND	BF	ND	BF	BF	ND	36.5	ND	ND	ND	7.0	1.1
**hsa-miR-181a-3p**	35.2	34.7	36.8	ND	34.6	34.6	35.7	37.0	ND	ND	BF	BF	ND	BF	BF	ND	ND	ND	7.0	1.0
**hsa-miR-30c-2-3p**	BF	35.9	36.4	ND	36.5	36.6	36.5	34.8	34.7	ND	ND	ND	ND	ND	ND	ND	ND	ND	7.0	0.8
**hsa-miR-570-3p**	36.8	36.1	36.2	ND	35.1	36.6	ND	35.6	ND	ND	36.6	ND	ND	ND	ND	ND	ND	ND	7.0	0.6
**hsa-miR-200c-3p**	35.9	ND	36.5	36.5	ND	ND	ND	35.3	ND	ND	ND	35.8	ND	ND	35.8	36.5	ND	ND	7.0	0.5
**hsa-miR-219-5p**	35.9	BF	36.6	35.8	35.9	35.6	ND	BF	ND	ND	BF	36.1	ND	BF	37.0	BF	ND	BF	7.0	0.5
**hsa-miR-628-3p**	36.7	36.9	36.2	BF	35.9	ND	36.2	35.7	BF	ND	ND	BF	ND	36.5	ND	ND	ND	BF	7.0	0.4
**hsa-miR-374b-3p**	36.3	35.9	36.2	ND	36.5	BF	36.0	ND	36.3	ND	ND	ND	ND	ND	BF	ND	ND	36.9	7.0	0.3
**hsa-miR-940**	ND	ND	ND	ND	ND	ND	30.1	ND	30.7	ND	36.7	34.5	ND	36.7	ND	ND	ND	36.2	6.0	3.0
**hsa-miR-142-5p**	BF	ND	ND	ND	ND	ND	ND	ND	ND	BF	35.8	34.3	34.6	33.9	32.5	BF	BF	34.9	6.0	1.1
**hsa-miR-124-3p**	34.7	ND	35.3	36.7	34.6	35.3	ND	36.4	ND	ND	ND	ND	BF	ND	ND	ND	ND	ND	6.0	0.9
**hsa-miR-338-3p**	ND	ND	ND	ND	ND	ND	ND	BF	ND	ND	35.5	36.3	35.7	35.6	34.0	ND	BF	36.4	6.0	0.9
**hsa-miR-625-3p**	35.0	35.1	36.4	BF	BF	35.5	36.7	ND	BF	BF	ND	36.7	ND	BF	BF	ND	ND	BF	6.0	0.8
**hsa-miR-34b-3p**	35.8	BF	ND	36.1	34.7	35.9	35.6	36.4	ND	ND	ND	ND	ND	ND	ND	ND	BF	ND	6.0	0.6
**hsa-miR-369-5p**	35.9	BF	35.7	ND	ND	36.6	35.8	36.5	BF	ND	ND	ND	ND	ND	36.5	ND	ND	BF	6.0	0.4
**hsa-miR-885-5p**	ND	ND	ND	ND	ND	ND	ND	ND	ND	ND	36.5	36.2	36.0	35.9	35.7	36.0	ND	BF	6.0	0.3
**hsa-miR-203a**	ND	ND	ND	ND	36.7	ND	ND	ND	ND	ND	35.9	ND	35.2	BF	34.9	ND	36.0	BF	5.0	0.7
**hsa-miR-181c-5p**	36.8	ND	BF	BF	ND	35.6	ND	35.1	ND	ND	ND	ND	ND	ND	36.5	ND	ND	35.7	5.0	0.7
**hsa-miR-495-3p**	30.6	30.8	30.7	BF	30.7	30.1	BF	BF	BF	BF	BF	BF	BF	BF	BF	BF	BF	BF	5.0	0.3
**hsa-miR-129-5p**	ND	ND	36.8	36.9	36.8	ND	35.2	ND	ND	ND	ND	ND	ND	ND	ND	ND	ND	ND	4.0	0.8
**hsa-miR-299-3p**	BF	36.8	36.2	ND	35.1	BF	ND	36.0	ND	ND	ND	ND	ND	ND	ND	ND	ND	ND	4.0	0.7
**hsa-miR-373-3p**	36.8	ND	BF	35.7	ND	36.0	ND	ND	ND	ND	ND	ND	ND	ND	37.0	ND	ND	ND	4.0	0.6
**hsa-miR-33b-5p**	35.7	ND	ND	ND	36.0	36.7	36.5	ND	ND	ND	ND	ND	ND	ND	ND	ND	ND	ND	4.0	0.5

Supplementary data

**hsa-miR-200a-3p**	ND	ND	ND	ND	ND	36.4	ND	ND	ND	ND	ND	36.5	35.8	36.6	ND	ND	ND	ND	4.0	0.4
**hsa-miR-887**	36.1	BF	BF	BF	36.3	ND	BF	36.7	36.1	ND	ND	ND	ND	ND	ND	ND	ND	BF	4.0	0.3
**hsa-miR-934**	BF	37.0	36.9	BF	ND	BF	BF	36.9	ND	BF	ND	BF	ND	BF	36.6	BF	BF	BF	4.0	0.2
**hsa-miR-134**	31.2	31.5	BF	BF	31.4	31.2	BF	BF	BF	BF	BF	BF	BF	BF	BF	BF	BF	BF	4.0	0.2
**hsa-miR-582-5p**	ND	BF	ND	36.4	ND	36.5	ND	ND	ND	36.6	ND	36.5	BF	ND	ND	ND	ND	ND	4.0	0.1
**hsa-miR-141-3p**	36.8	35.6	ND	ND	ND	BF	ND	ND	ND	ND	ND	BF	37.0	ND	BF	BF	ND	ND	3.0	0.7
**hsa-miR-135a-5p**	36.5	ND	BF	BF	ND	BF	ND	ND	ND	BF	BF	36.0	ND	ND	BF	36.9	ND	ND	3.0	0.5
**hsa-miR-302d-3p**	ND	ND	ND	ND	ND	36.4	ND	36.6	ND	ND	ND	ND	ND	ND	ND	ND	ND	36.8	3.0	0.2
**hsa-miR-576-3p**	35.1	36.8	ND	BF	BF	ND	BF	BF	ND	ND	ND	ND	ND	ND	ND	ND	ND	ND	2.0	1.2
**hsa-miR-187-3p**	ND	ND	36.6	ND	ND	ND	ND	35.6	ND	ND	ND	ND	ND	ND	ND	ND	ND	ND	2.0	0.7
**hsa-miR-499a-5p**	BF	ND	ND	ND	ND	ND	ND	ND	ND	ND	36.9	BF	ND	ND	36.0	ND	ND	ND	2.0	0.6
**hsa-miR-651**	36.0	ND	ND	ND	ND	ND	36.7	ND	ND	ND	ND	ND	BF	ND	ND	ND	ND	ND	2.0	0.5
**hsa-miR-198**	36.9	ND	ND	BF	36.6	ND	ND	ND	ND	ND	ND	ND	ND	ND	ND	ND	ND	ND	2.0	0.2
**hsa-miR-187-5p**	ND	36.8	ND	ND	ND	ND	ND	ND	ND	BF	BF	ND	ND	BF	36.6	ND	ND	ND	2.0	0.2
**hsa-miR-549a**	36.8	BF	ND	ND	BF	36.6	BF	ND	ND	ND	ND	ND	ND	ND	ND	ND	ND	BF	2.0	0.2
**hsa-miR-551b-3p**	ND	ND	ND	ND	36.7	ND	ND	ND	ND	ND	ND	ND	36.6	ND	BF	ND	ND	ND	2.0	0.1
**hsa-miR-345-5p**	BF	BF	BF	36.7	ND	36.6	ND	ND	BF	ND	ND	ND	ND	ND	ND	ND	ND	ND	2.0	0.1
**hsa-miR-96-5p**	ND	ND	ND	ND	ND	36.7	ND	ND	ND	ND	ND	ND	ND	ND	ND	ND	ND	ND	1.0	ND
**hsa-miR-9-5p**	ND	ND	ND	ND	ND	ND	ND	ND	ND	ND	ND	ND	ND	ND	36.4	ND	ND	ND	1.0	ND
**hsa-miR-760**	ND	BF	ND	ND	ND	ND	ND	ND	ND	ND	ND	ND	ND	ND	34.7	ND	ND	ND	1.0	ND
**hsa-miR-662**	ND	ND	BF	ND	ND	36.8	ND	ND	ND	ND	ND	ND	BF	ND	ND	ND	ND	ND	1.0	ND
**hsa-miR-627**	35.9	ND	ND	BF	ND	BF	ND	ND	ND	ND	ND	ND	ND	ND	ND	ND	ND	ND	1.0	ND
**hsa-miR-545-3p**	ND	BF	ND	BF	BF	36.4	ND	ND	ND	ND	ND	ND	ND	ND	ND	ND	ND	ND	1.0	ND
**hsa-miR-519a-3p**	ND	ND	ND	ND	ND	ND	ND	ND	ND	ND	ND	ND	ND	37.0	ND	ND	ND	ND	1.0	ND
**hsa-miR-518c-3p**	ND	ND	ND	ND	ND	ND	ND	ND	ND	ND	ND	ND	ND	ND	ND	ND	ND	36.7	1.0	ND
**hsa-miR-509-3p**	ND	ND	ND	ND	ND	ND	ND	ND	36.3	ND	ND	ND	ND	ND	BF	ND	ND	ND	1.0	ND
**hsa-miR-483-3p**	ND	ND	ND	ND	ND	ND	ND	ND	34.2	ND	BF	BF	BF	BF	ND	ND	ND	BF	1.0	ND
**hsa-miR-383**	36.6	ND	ND	ND	ND	ND	ND	ND	ND	ND	ND	ND	ND	ND	ND	ND	ND	ND	1.0	ND
**hsa-miR-375**	ND	ND	ND	ND	ND	ND	ND	ND	ND	ND	ND	ND	ND	ND	ND	ND	ND	35.6	1.0	ND
**hsa-miR-373-5p**	ND	ND	ND	ND	ND	BF	ND	ND	ND	ND	ND	ND	ND	BF	36.1	ND	ND	ND	1.0	ND
**hsa-miR-372**	35.7	ND	ND	ND	ND	ND	ND	ND	ND	ND	ND	ND	ND	ND	ND	ND	ND	ND	1.0	ND
**hsa-miR-362-5p**	BF	BF	BF	BF	33.8	BF	BF	BF	BF	BF	BF	BF	BF	BF	BF	BF	BF	BF	1.0	ND
**hsa-miR-34c-3p**	ND	ND	ND	ND	ND	ND	ND	ND	ND	ND	ND	ND	ND	36.7	ND	BF	ND	ND	1.0	ND

Supplementary data

**hsa-miR-302b-3p**	ND	ND	BF	ND	ND	ND	ND	ND	36.9	ND	ND	ND	ND	BF	ND	ND	ND	ND	1.0	ND
**hsa-miR-302a-3p**	ND	ND	36.9	ND	ND	ND	ND	ND	ND	ND	ND	ND	ND	ND	ND	ND	ND	ND	1.0	ND
**hsa-miR-205-5p**	ND	ND	ND	ND	ND	ND	ND	ND	ND	ND	ND	ND	ND	ND	ND	ND	ND	35.7	1.0	ND
**hsa-miR-184**	ND	ND	ND	ND	ND	ND	ND	36.7	BF	ND	ND	ND	ND	ND	ND	ND	ND	ND	1.0	ND
**hsa-miR-153**	ND	36.8	ND	BF	ND	BF	ND	ND	ND	ND	ND	ND	ND	ND	ND	ND	ND	BF	1.0	ND
**hsa-miR-147b**	ND	BF	ND	ND	ND	ND	ND	ND	ND	ND	ND	ND	ND	ND	ND	ND	ND	36.7	1.0	ND
**hsa-miR-1**	ND	ND	ND	ND	BF	ND	ND	ND	BF	ND	ND	BF	ND	ND	37.0	ND	ND	BF	1.0	ND
**hsa-miR-96-3p**	ND	ND	ND	ND	BF	ND	ND	ND	ND	ND	ND	ND	ND	ND	ND	ND	ND	ND	0.0	ND
**hsa-miR-9-3p**	ND	ND	ND	BF	ND	ND	ND	ND	ND	ND	ND	ND	ND	ND	ND	BF	ND	ND	0.0	ND
**hsa-miR-933**	ND	BF	ND	ND	ND	ND	ND	ND	ND	ND	ND	ND	ND	ND	ND	ND	ND	ND	0.0	ND
**hsa-miR-765**	ND	ND	ND	ND	ND	ND	ND	ND	ND	ND	ND	ND	ND	ND	ND	ND	ND	ND	0.0	ND
**hsa-miR-668**	ND	ND	ND	ND	ND	BF	ND	BF	ND	ND	ND	ND	ND	ND	ND	ND	ND	ND	0.0	ND
**hsa-miR-663a**	ND	ND	ND	BF	ND	ND	ND	ND	BF	ND	ND	BF	BF	ND	ND	ND	BF	BF	0.0	ND
**hsa-miR-598**	BF	BF	BF	BF	BF	BF	ND	BF	BF	ND	ND	BF	BF	ND	BF	ND	ND	ND	0.0	ND
**hsa-miR-597**	ND	ND	BF	ND	ND	ND	ND	ND	ND	ND	ND	ND	ND	ND	ND	ND	ND	ND	0.0	ND
**hsa-miR-596**	ND	ND	ND	ND	ND	ND	BF	ND	ND	ND	ND	ND	ND	ND	ND	ND	ND	ND	0.0	ND
**hsa-miR-589-5p**	ND	BF	ND	ND	BF	BF	BF	BF	ND	ND	ND	ND	ND	ND	ND	ND	ND	ND	0.0	ND
**hsa-miR-572**	ND	ND	ND	ND	ND	BF	ND	ND	ND	ND	ND	ND	ND	ND	ND	ND	ND	ND	0.0	ND
**hsa-miR-510**	ND	ND	BF	ND	ND	ND	ND	ND	ND	ND	ND	ND	ND	ND	ND	ND	ND	ND	0.0	ND
**hsa-miR-509-3-5p**	ND	ND	ND	ND	ND	ND	BF	ND	ND	ND	ND	ND	ND	ND	ND	ND	ND	ND	0.0	ND
**hsa-miR-508-3p**	ND	ND	ND	ND	ND	ND	ND	ND	BF	ND	ND	ND	ND	ND	ND	ND	ND	ND	0.0	ND
**hsa-miR-502-5p**	ND	ND	ND	ND	ND	ND	ND	ND	ND	ND	ND	ND	BF	ND	ND	ND	ND	ND	0.0	ND
**hsa-miR-490-3p**	BF	BF	BF	BF	BF	BF	BF	BF	BF	BF	BF	BF	BF	BF	BF	BF	BF	BF	0.0	ND
**hsa-miR-449a**	ND	ND	ND	ND	BF	ND	BF	ND	BF	ND	ND	ND	ND	ND	ND	ND	ND	ND	0.0	ND
**hsa-miR-422a**	ND	ND	ND	ND	ND	ND	ND	ND	ND	ND	BF	ND	ND	ND	ND	ND	ND	ND	0.0	ND
**hsa-miR-371a-5p**	BF	ND	ND	ND	ND	ND	ND	ND	ND	ND	ND	ND	ND	ND	BF	ND	ND	ND	0.0	ND
**hsa-miR-367-3p**	ND	ND	ND	ND	ND	ND	ND	ND	ND	ND	ND	ND	ND	ND	ND	ND	ND	ND	0.0	ND
**hsa-miR-363-3p**	ND	ND	ND	ND	ND	ND	ND	BF	ND	ND	ND	ND	ND	ND	BF	ND	ND	ND	0.0	ND
**hsa-miR-346**	BF	BF	ND	BF	ND	ND	ND	BF	ND	ND	BF	ND	ND	BF	BF	BF	BF	ND	0.0	ND
**hsa-miR-329**	BF	BF	BF	BF	BF	BF	BF	BF	BF	BF	BF	BF	BF	BF	BF	BF	BF	BF	0.0	ND
**hsa-miR-302c-5p**	ND	ND	ND	ND	ND	ND	ND	ND	ND	ND	ND	ND	ND	ND	ND	ND	ND	ND	0.0	ND
**hsa-miR-26a-2-3p**	ND	BF	BF	BF	ND	BF	ND	ND	ND	ND	ND	ND	ND	ND	ND	ND	ND	ND	0.0	ND
**hsa-miR-216a-5p**	ND	ND	ND	BF	ND	ND	ND	ND	ND	ND	ND	ND	ND	ND	ND	ND	ND	ND	0.0	ND

Supplementary data

**hsa-miR-206**	BF	ND	BF	BF	ND	BF	ND	BF	BF	BF	BF	BF	BF	BF	BF	BF	BF	ND	0.0	ND
**hsa-miR-200b-3p**	ND	ND	ND	ND	ND	ND	ND	BF	ND	ND	ND	BF	ND	ND	ND	ND	ND	BF	0.0	ND
**hsa-miR-185-3p**	ND	ND	ND	ND	ND	BF	ND	ND	BF	ND	ND	ND	ND	ND	ND	ND	ND	ND	0.0	ND
**hsa-miR-16-1-3p**	BF	ND	BF	BF	BF	ND	BF	BF	ND	BF	BF	ND	ND	BF	BF	BF	ND	BF	0.0	ND
**hsa-miR-129-2-3p**	ND	ND	ND	ND	ND	BF	ND	ND	ND	ND	ND	ND	ND	ND	ND	ND	ND	ND	0.0	ND
**hsa-miR-128**	BF	BF	BF	BF	BF	BF	BF	BF	BF	BF	BF	BF	BF	BF	BF	BF	BF	BF	0.0	ND
**Total counts**	220	206	208	200	212	218	200	204	189	143	146	179	156	184	208	134	108	178		
