# Supplementary material for: Mesenchymal stem cell-derived exosomes have altered microRNA profiles and induce osteogenic differentiation depending on the stage of differentiation
Source: PLoS One. 2018 Feb 15;13(2):e0193059. doi: 10.1371/journal.pone.0193059 (PMC5814093; doi:10.1371/journal.pone.0193059)
Supplement: S2 Table — (PDF) [file pone.0193059.s005.pdf]

Supplementary data

S2 Table. Top 50 expressed microRNAs based on mean of normalized dcq value

microRNAs \ groups	hMSC_P6	hMSC_D3	hMSC_D21	Exo_P6	Exo_D3	Exo_D21
	hsa-miR-125b-5p	hsa-miR-125b-5p	hsa-let-7a-5p	hsa-miR-21-5p	hsa-miR-21-5p	hsa-miR-21-5p
	hsa-miR-21-5p	hsa-miR-21-5p	hsa-miR-125b-5p	hsa-miR-125b-5p	hsa-miR-125b-5p	hsa-miR-16-5p
	hsa-let-7a-5p	hsa-let-7a-5p	hsa-miR-21-5p	hsa-miR-221-3p	hsa-miR-16-5p	hsa-miR-125b-5p
	hsa-miR-221-3p	hsa-miR-221-3p	hsa-miR-221-3p	hsa-miR-16-5p	hsa-miR-221-3p	hsa-miR-126-3p
	hsa-miR-16-5p	hsa-miR-199a-3p	hsa-let-7b-5p	hsa-let-7a-5p	hsa-miR-23a-3p	hsa-let-7a-5p
	hsa-miR-100-5p	hsa-miR-16-5p	hsa-miR-145-5p	hsa-miR-23a-3p	hsa-let-7a-5p	hsa-miR-23a-3p
	hsa-miR-199a-3p	hsa-miR-23a-3p	hsa-miR-23a-3p	hsa-miR-100-5p	hsa-miR-126-3p	hsa-miR-221-3p
	hsa-miR-23a-3p	hsa-miR-100-5p	hsa-miR-24-3p	hsa-miR-142-3p	hsa-miR-451a	hsa-miR-142-3p
	hsa-miR-31-5p	hsa-miR-24-3p	hsa-miR-199a-3p	hsa-miR-222-3p	hsa-miR-100-5p	hsa-miR-24-3p
	hsa-miR-222-3p	hsa-let-7b-5p	hsa-miR-125a-5p	hsa-miR-24-3p	hsa-miR-142-3p	hsa-miR-100-5p
	hsa-miR-24-3p	hsa-miR-222-3p	hsa-let-7i-5p	hsa-miR-451a	hsa-miR-222-3p	hsa-miR-223-3p
	hsa-let-7i-5p	hsa-let-7i-5p	hsa-miR-100-5p	hsa-miR-223-3p	hsa-miR-24-3p	hsa-miR-451a
	hsa-let-7b-5p	hsa-miR-199a-5p	hsa-miR-16-5p	hsa-miR-126-3p	hsa-miR-223-3p	hsa-miR-199a-3p
	hsa-miR-29a-3p	hsa-miR-125a-5p	hsa-let-7e-5p	hsa-miR-199a-3p	hsa-miR-199a-3p	hsa-miR-26a-5p
	hsa-miR-34a-5p	hsa-miR-31-5p	hsa-miR-222-3p	hsa-miR-92a-3p	hsa-miR-92a-3p	hsa-miR-27b-3p
	hsa-miR-26a-5p	hsa-miR-34a-5p	hsa-miR-26a-5p	hsa-miR-31-5p	hsa-miR-26a-5p	hsa-miR-92a-3p
	hsa-miR-125a-5p	hsa-miR-26a-5p	hsa-miR-214-3p	hsa-miR-26a-5p	hsa-miR-20a-5p	hsa-miR-145-5p
	hsa-miR-103a-3p	hsa-miR-29a-3p	hsa-let-7c	hsa-miR-20a-5p	hsa-miR-19b-3p	hsa-miR-23b-3p
	hsa-miR-199a-5p	hsa-miR-103a-3p	hsa-miR-34a-5p	hsa-miR-106a-5p	hsa-miR-27b-3p	hsa-miR-222-3p
	hsa-let-7c	hsa-miR-214-3p	hsa-miR-103a-3p	hsa-miR-27b-3p	hsa-miR-23b-3p	hsa-miR-20a-5p
	hsa-miR-199b-5p	hsa-miR-22-3p	hsa-miR-193b-3p	hsa-miR-23b-3p	hsa-miR-145-5p	hsa-let-7b-5p
	hsa-miR-15a-5p	hsa-let-7c	hsa-miR-23b-3p	hsa-miR-103a-3p	hsa-miR-15a-5p	hsa-miR-103a-3p
	hsa-let-7e-5p	hsa-let-7e-5p	hsa-miR-199a-5p	hsa-miR-19b-3p	hsa-miR-103a-3p	hsa-miR-106a-5p
	hsa-let-7g-5p	hsa-let-7g-5p	hsa-miR-22-3p	hsa-miR-145-5p	hsa-miR-106a-5p	hsa-miR-125a-5p
	hsa-miR-23b-3p	hsa-miR-193b-3p	hsa-let-7g-5p	hsa-let-7b-5p	hsa-miR-31-5p	hsa-miR-10b-5p
	hsa-miR-214-3p	hsa-miR-199b-5p	hsa-miR-181a-5p	hsa-miR-93-5p	hsa-let-7b-5p	hsa-miR-152
	hsa-miR-22-3p	hsa-miR-23b-3p	hsa-miR-27b-3p	hsa-miR-19a-3p	hsa-miR-93-5p	hsa-miR-181a-5p
	hsa-miR-29c-3p	hsa-miR-145-5p	hsa-miR-127-3p	hsa-miR-15a-5p	hsa-miR-125a-5p	hsa-miR-150-5p
	hsa-miR-20a-5p	hsa-miR-92a-3p	hsa-miR-29a-3p	hsa-let-7i-5p	hsa-miR-29a-3p	hsa-miR-93-5p
	hsa-miR-19b-3p	hsa-miR-19b-3p	hsa-miR-320a	hsa-miR-29a-3p	hsa-miR-19a-3p	hsa-miR-15a-5p
	hsa-miR-92a-3p	hsa-miR-15a-5p	hsa-let-7d-5p	hsa-miR-125a-5p	hsa-let-7g-5p	hsa-let-7g-5p
	hsa-miR-27b-3p	hsa-miR-27b-3p	hsa-miR-92a-3p	hsa-let-7g-5p	hsa-let-7i-5p	hsa-let-7e-5p
	hsa-miR-145-5p	hsa-miR-20a-5p	hsa-miR-31-5p	hsa-miR-486-5p	hsa-miR-181a-5p	hsa-miR-214-3p
	hsa-miR-193b-3p	hsa-miR-127-3p	hsa-miR-423-3p	hsa-miR-150-5p	hsa-miR-10b-5p	hsa-let-7i-5p
	hsa-miR-152	hsa-miR-152	hsa-miR-199b-5p	hsa-miR-152	hsa-miR-150-5p	hsa-miR-361-5p
	hsa-miR-127-3p	hsa-miR-106a-5p	hsa-let-7f-5p	hsa-miR-27a-3p	hsa-miR-199b-5p	hsa-miR-486-5p
	hsa-miR-106a-5p	hsa-miR-29c-3p	hsa-miR-143-3p	hsa-miR-199b-5p	hsa-miR-152	hsa-miR-151a-5p
	hsa-miR-107	hsa-miR-27a-3p	hsa-miR-99b-5p	hsa-let-7c	hsa-miR-25-3p	hsa-let-7c
	hsa-miR-423-3p	hsa-miR-10b-5p	hsa-miR-152	hsa-miR-151a-5p	hsa-miR-486-5p	hsa-miR-25-3p
	hsa-miR-181a-5p	hsa-miR-423-3p	hsa-miR-107	hsa-miR-10b-5p	hsa-miR-27a-3p	hsa-miR-19b-3p
	hsa-miR-29b-3p	hsa-miR-320a	hsa-miR-574-3p	hsa-miR-181a-5p	hsa-let-7c	hsa-miR-31-5p
	hsa-miR-320a	hsa-miR-107	hsa-miR-365a-3p	hsa-miR-25-3p	hsa-miR-34a-5p	hsa-miR-199b-5p
	hsa-miR-151a-5p	hsa-let-7f-5p	hsa-miR-27a-3p	hsa-miR-214-3p	hsa-miR-214-3p	hsa-miR-30c-5p
	hsa-miR-93-5p	hsa-miR-19a-3p	hsa-miR-423-5p	hsa-miR-30c-5p	hsa-miR-320a	hsa-miR-409-3p
	hsa-miR-19a-3p	hsa-miR-365a-3p	hsa-miR-151a-5p	hsa-miR-320a	hsa-let-7e-5p	hsa-miR-191-5p
	hsa-miR-27a-3p	hsa-miR-181a-5p	hsa-miR-197-3p	hsa-miR-423-5p	hsa-miR-122-5p	hsa-miR-27a-3p
	hsa-let-7f-5p	hsa-let-7d-5p	hsa-miR-361-5p	hsa-miR-122-5p	hsa-miR-30c-5p	hsa-miR-99a-5p
	hsa-miR-99b-5p	hsa-miR-151a-5p	hsa-miR-191-5p	hsa-miR-15b-5p	hsa-miR-127-3p	hsa-miR-127-3p
	hsa-miR-31-3p	hsa-miR-361-5p	hsa-miR-29c-3p	hsa-miR-193b-3p	hsa-miR-99a-5p	hsa-miR-320a
	hsa-miR-361-5p	hsa-miR-136-5p	hsa-miR-409-3p	hsa-miR-30b-5p	hsa-miR-106b-5p	hsa-miR-143-3p
