# Supplementary material for: Mesenchymal stem cell-derived exosomes have altered microRNA profiles and induce osteogenic differentiation depending on the stage of differentiation
Source: PLoS One. 2018 Feb 15;13(2):e0193059. doi: 10.1371/journal.pone.0193059 (PMC5814093; doi:10.1371/journal.pone.0193059)
Supplement: S5 Table — (PDF) [file pone.0193059.s008.pdf]

Supplementary data

S5 Table. Biological pathways enriched by differentially expressed microRNAs in Exo_D3 vs Exo_P6

KEGG pathway	p-value, FDR corrected	genes	miRNAs	algorithms
Hippo signaling pathway (hsa04390)	1.41E-05	9	1	microT-CDS
Glycosphingolipid biosynthesis - lacto and neolacto series (hsa00601)	6.59E-03	2	1	microT-CDS
Adherens junction (hsa04520)	6.59E-03	6	1	microT-CDS
Fatty acid biosynthesis (hsa00061)	1.23E-17	1	1	Tarbase
Fatty acid metabolism (hsa01212)	1.39E-06	4	1	Tarbase
ECM-receptor interaction (hsa04512)	5.63E-05	11	1	Tarbase
Adherens junction (hsa04520)	3.98E-04	15	1	Tarbase
Lysine degradation (hsa00310)	7.56E-04	11	1	Tarbase
Glycosaminoglycan biosynthesis - chondroitin sulfate / dermatan sulfate (hsa00532)	2.58E-03	4	1	Tarbase
Steroid biosynthesis (hsa00100)	4.44E-03	4	1	Tarbase
2-Oxocarboxylic acid metabolism(hsa01210)	9.67E-03	4	1	Tarbase
Biosynthesis of amino acids (hsa01230)	9.67E-03	14	1	Tarbase
Ribosome (hsa03010)	9.67E-03	26	1	Tarbase
Bacterial invasion of epithelial cells (hsa05100)	9.67E-03	15	1	Tarbase
Pathogenic Escherichia coli infection (hsa05130)	9.67E-03	14	1	Tarbase
Central carbon metabolism in cancer (hsa05230)	1.52E-02	10	1	Tarbase
Fatty acid elongation (hsa00062)	1.53E-02	2	1	Tarbase
Cell cycle (hsa04110)	2.85E-02	22	1	Tarbase
Proteoglycans in cancer (hsa05205)	3.47E-02	27	1	Tarbase
Spliceosome (hsa03040)	4.21E-02	22	1	Tarbase
Hippo signaling pathway (hsa04390)	4.21E-02	16	1	Tarbase
