# Supplementary material for: Mesenchymal stem cell-derived exosomes have altered microRNA profiles and induce osteogenic differentiation depending on the stage of differentiation
Source: PLoS One. 2018 Feb 15;13(2):e0193059. doi: 10.1371/journal.pone.0193059 (PMC5814093; doi:10.1371/journal.pone.0193059)
Supplement: S6 Table — (PDF) [file pone.0193059.s009.pdf]

Supplementary data

S6 Table. Biological pathways enriched by differentially expressed microRNAs in Exo_D21 vs Exo_P6

KEGG pathway	p-value, FDR corrected	genes	miRNAs	algorithms
ECM-receptor interaction (hsa04512)	8.84E-63	40	8	microT-CDS
Prion diseases (hsa05020)	1.64E-09	5	5	microT-CDS
Amoebiasis (hsa05146)	4.30E-09	43	9	microT-CDS
Proteoglycans in cancer (hsa05205)	1.66E-08	66	9	microT-CDS
Glioma (hsa05214)	3.86E-07	28	8	microT-CDS
Focal adhesion (hsa04510)	7.16E-07	79	9	microT-CDS
Renal cell carcinoma (hsa05211)	2.31E-05	30	8	microT-CDS
ErbB signaling pathway (hsa04012)	2.12E-04	36	9	microT-CDS
PI3K-Akt signaling pathway (hsa04151)	2.12E-04	104	9	microT-CDS
Choline metabolism in cancer (hsa05231)	2.70E-04	40	8	microT-CDS
Non-small cell lung cancer (hsa05223)	2.75E-04	24	8	microT-CDS
Lysine degradation (hsa00310)	8.02E-04	16	7	microT-CDS
Estrogen signaling pathway (hsa04915)	1.30E-03	32	9	microT-CDS
Ras signaling pathway (hsa04014)	2.27E-03	65	9	microT-CDS
Signaling pathways regulating pluripotency of stem cells (hsa04550)	2.66E-03	46	9	microT-CDS
Platelet activation (hsa04611)	3.78E-03	41	9	microT-CDS
Rap1 signaling pathway (hsa04015)	4.48E-03	65	9	microT-CDS
FoxO signaling pathway (hsa04068)	1.49E-02	44	8	microT-CDS
Morphine addiction (hsa05032)	1.49E-02	30	9	microT-CDS
Protein digestion and absorption (hsa04974)	1.51E-02	32	8	microT-CDS
Small cell lung cancer (hsa05222)	1.63E-02	31	9	microT-CDS
Neurotrophin signaling pathway (hsa04722)	1.68E-02	41	9	microT-CDS
MAPK signaling pathway	1.85E-02	74	9	microT-CDS
Thyroid hormone signaling pathway (hsa04010)	1.90E-02	35	7	microT-CDS
Wnt signaling pathway (hsa04310)	2.23E-02	41	9	microT-CDS
Glycosaminoglycan biosynthesis - keratan sulfate (hsa00533)	2.58E-02	5	4	microT-CDS
Amphetamine addiction (hsa05031)	2.58E-02	23	8	microT-CDS
mTOR signaling pathway (hsa04150)	2.65E-02	23	7	microT-CDS
Gap junction (hsa04540)	2.65E-02	29	8	microT-CDS
T cell receptor signaling pathway (hsa04660)	3.12E-02	34	9	microT-CDS
Endometrial cancer (hsa05213)	3.19E-02	20	7	microT-CDS
Aldosterone-regulated sodium reabsorption (hsa04960)	3.23E-02	16	6	microT-CDS
Transcriptional misregulation in cancer (hsa05202)	3.67E-02	52	9	microT-CDS
Regulation of actin cytoskeleton (hsa04810)	3.92E-02	65	9	microT-CDS
Dorso-ventral axis formation (hsa04320)	4.14E-02	12	6	microT-CDS
cAMP signaling pathway (hsa04024)	4.14E-02	58	9	microT-CDS

Supplementary data

S6 Table cont. Biological pathways enriched by differentially expressed microRNAs in Exo_D21 vs Exo_P6

KEGG pathway	p-value, FDR corrected	genes	miRNAs	algorithms
Hippo signaling pathway (hsa04390)	4.14E-02	37	9	microT-CDS
Cholinergic synapse (hsa04725)	4.14E-02	34	9	microT-CDS
Fc epsilon RI signaling pathway (hsa04664)	4.47E-02	25	7	microT-CDS
Prostate cancer (hsa05215)	4.58E-02	29	8	microT-CDS
Sphingolipid signaling pathway (hsa04071)	4.90E-02	33	8	microT-CDS
Proteoglycans in cancer (hsa05205)	1.22E-17	112	9	Tarbase
Viral carcinogenesis (hsa05203)	2.40E-12	110	9	Tarbase
Chronic myeloid leukemia (hsa05220)	5.32E-10	52	9	Tarbase
Hepatitis B (hsa05161)	1.16E-09	81	9	Tarbase
Protein processing in endoplasmic reticulum (hsa04141)	4.76E-08	94	9	Tarbase
Pancreatic cancer (hsa05212)	1.10E-07	44	9	Tarbase
Hippo signaling pathway (hsa04390)	1.35E-07	74	8	Tarbase
TGF-beta signaling pathway (hsa04350)	1.35E-07	45	9	Tarbase
Adherens junction (hsa04520)	3.29E-07	45	9	Tarbase
Glioma (hsa05214)	4.33E-07	40	9	Tarbase
Colorectal cancer (hsa05210)	6.46E-07	42	9	Tarbase
ECM-receptor interaction (mmu04512)	8.27E-07	36	9	Tarbase
Prion diseases (hsa05020)	2.04E-06	12	8	Tarbase
Cell cycle (hsa04110)	4.52E-06	69	9	Tarbase
Non-small cell lung cancer (hsa05223)	1.11E-05	34	9	Tarbase
Signaling pathways regulating pluripotency of stem cells (hsa04550)	1.38E-05	73	9	Tarbase
Estrogen signaling pathway (hsa04915)	1.38E-05	54	9	Tarbase
Renal cell carcinoma (hsa05211)	1.53E-05	42	8	Tarbase
Focal adhesion (mmu04510)	1.53E-05	110	9	Tarbase
Ubiquitin mediated proteolysis (hsa04120)	2.06E-05	79	9	Tarbase
Thyroid hormone signaling pathway (hsa04919)	2.06E-05	67	9	Tarbase
Bacterial invasion of epithelial cells (hsa05100)	3.17E-05	45	9	Tarbase
Central carbon metabolism in cancer (hsa05230)	3.78E-05	39	9	Tarbase
Oocyte meiosis (hsa04114)	9.23E-05	57	9	Tarbase
Neurotrophin signaling pathway (hsa04722)	9.88E-05	66	9	Tarbase
Fatty acid biosynthesis (hsa00061)	1.18E-04	4	7	Tarbase
Sphingolipid signaling pathway (hsa04071)	1.46E-04	61	9	Tarbase
Pathways in cancer (hsa05200)	1.55E-04	184	9	Tarbase
mTOR signaling pathway (hsa04150)	1.90E-04	38	9	Tarbase
Endometrial cancer (hsa05213)	1.90E-04	32	9	Tarbase
FoxO signaling pathway (hsa04068)	2.37E-04	74	9	Tarbase

Supplementary data

S6 Table cont. Biological pathways enriched by differentially expressed microRNAs in Exo_D21 vs Exo_P6

KEGG pathway	p-value, FDR corrected	genes	miRNAs	algorithms
Epstein-Barr virus infection (hsa05169)	2.37E-04	101	9	Tarbase
Progesterone-mediated oocyte maturation (hsa04914)	2.52E-04	51	9	Tarbase
Prolactin signaling pathway (hsa04917)	2.69E-04	39	8	Tarbase
HTLV-I infection (hsa05166)	2.71E-04	126	9	Tarbase
p53 signaling pathway (hsa04115)	3.53E-04	41	9	Tarbase
ErbB signaling pathway (hsa04012)	5.63E-04	48	9	Tarbase
Small cell lung cancer (hsa05222)	8.86E-04	48	9	Tarbase
Prostate cancer (hsa05215)	1.10E-03	50	9	Tarbase
Bladder cancer (hsa05219)	1.53E-03	25	9	Tarbase
TNF signaling pathway (hsa04668)	2.05E-03	55	9	Tarbase
HIF-1 signaling pathway (hsa04066)	3.13E-03	56	9	Tarbase
Lysine degradation (hsa00310)	3.59E-03	24	8	Tarbase
Steroid biosynthesis (hsa00100)	4.53E-03	11	8	Tarbase
Melanoma (hsa05218)	5.43E-03	36	9	Tarbase
AMPK signaling pathway (hsa04152)	5.94E-03	62	9	Tarbase
Shigellosis (hsa05131)	6.06E-03	34	8	Tarbase
Wnt signaling pathway (hsa04310)	8.51E-03	66	9	Tarbase
Gap junction (hsa04540)	1.05E-02	43	9	Tarbase
Epithelial cell signaling in Helicobacter pylori infection (hsa05120)	1.44E-02	36	9	Tarbase
Platelet activation (hsa04611)	1.70E-02	61	9	Tarbase
Acute myeloid leukemia (hsa05221)	1.76E-02	31	8	Tarbase
Thyroid cancer (hsa05216)	1.78E-02	17	8	Tarbase
Sulfur metabolism (hsa00920)	2.26E-02	5	5	Tarbase
Insulin signaling pathway (hsa04910)	2.29E-02	67	9	Tarbase
Endocytosis (hsa04144)	2.69E-02	96	9	Tarbase
VEGF signaling pathway (hsa04370)	3.66E-02	32	9	Tarbase
Glycosaminoglycan biosynthesis - keratan sulfate (hsa00533)	3.79E-02	7	7	Tarbase
mRNA surveillance pathway (hsa03015)	3.95E-02	47	9	Tarbase
Circadian rhythm (hsa04710)	3.95E-02	18	9	Tarbase
Pathogenic Escherichia coli infection (hsa05130)	4.42E-02	29	9	Tarbase
Chagas disease (American trypanosomiasis) (hsa05142)	4.42E-02	49	9	Tarbase
Transcriptional misregulation in cancer (hsa05202)	4.45E-02	73	9	Tarbase
Apoptosis (hsa04210)	4.84E-02	43	9	Tarbase
Leukocyte transendothelial migration (hsa04670)	4.95E-02	54	9	Tarbase
