# Supplementary material for: Mesenchymal stem cell-derived exosomes have altered microRNA profiles and induce osteogenic differentiation depending on the stage of differentiation
Source: PLoS One. 2018 Feb 15;13(2):e0193059. doi: 10.1371/journal.pone.0193059 (PMC5814093; doi:10.1371/journal.pone.0193059)
Supplement: S7 Table — (PDF) [file pone.0193059.s010.pdf]

Supplementary data

S7 Table. Biological pathways enriched by differentially expressed microRNAs in Exo_D21 vs Exo_D3

KEGG pathway	p-value, FDR corrected	genes	miRNAs	algorithms
ECM-receptor interaction (hsa04512)	7.57E-35	44	14	microT-CDS
Fatty acid biosynthesis (hsa00061)	6.58E-15	8	6	microT-CDS
Proteoglycans in cancer (hsa05205)	6.58E-15	104	15	microT-CDS
Signaling pathways regulating pluripotency of stem cells (hsa04550)	4.86E-10	75	16	microT-CDS
Hippo signaling pathway (hsa04390)	1.11E-08	73	15	microT-CDS
Focal adhesion (hsa04510)	4.67E-08	107	17	microT-CDS
Pathways in cancer (hsa05200)	1.23E-07	173	16	microT-CDS
Glioma (hsa05214)	1.68E-07	36	15	microT-CDS
PI3K-Akt signaling pathway (hsa04151)	1.68E-07	155	16	microT-CDS
Prion diseases (hsa05020)	1.73E-07	10	9	microT-CDS
Ras signaling pathway (hsa04014)	2.74E-07	104	16	microT-CDS
Renal cell carcinoma (hsa05211)	2.78E-07	41	14	microT-CDS
Wnt signaling pathway (hsa04310)	4.85E-06	68	15	microT-CDS
Thyroid hormone signaling pathway (hsa04919)	8.14E-06	58	14	microT-CDS
TGF-beta signaling pathway (hsa04350)	1.67E-05	42	12	microT-CDS
Colorectal cancer (hsa05210)	1.67E-05	36	13	microT-CDS
mTOR signaling pathway (hsa04150)	1.67E-05	37	15	microT-CDS
MAPK signaling pathway (hsa04010)	1.67E-05	118	16	microT-CDS
Prostate cancer (hsa05215)	2.07E-05	48	15	microT-CDS
Melanoma (hsa05218)	3.23E-05	41	15	microT-CDS
FoxO signaling pathway (hsa04068)	3.23E-05	67	16	microT-CDS
Adrenergic signaling in cardiomyocytes (hsa04261)	4.23E-05	61	14	microT-CDS
Choline metabolism in cancer (hsa05231)	1.26E-04	51	14	microT-CDS
AMPK signaling pathway (hsa04152)	1.31E-04	62	15	microT-CDS
Amoebiasis (hsa05146)	1.73E-04	46	13	microT-CDS
Rap1 signaling pathway (hsa04015)	2.06E-04	94	15	microT-CDS
Non-small cell lung cancer (hsa05223)	2.06E-04	29	14	microT-CDS
Lysine degradation (hsa00310)	2.83E-04	21	10	microT-CDS
Prolactin signaling pathway (hsa04917)	4.40E-04	35	14	microT-CDS
Pancreatic cancer (hsa05212)	7.09E-04	34	14	microT-CDS
cAMP signaling pathway (hsa04024)	7.09E-04	88	15	microT-CDS
Neurotrophin signaling pathway (hsa04722)	7.09E-04	59	16	microT-CDS
Axon guidance (hsa04360)	8.41E-04	57	15	microT-CDS
Estrogen signaling pathway (hsa04915)	1.06E-03	43	15	microT-CDS
Small cell lung cancer (hsa05222)	1.11E-03	43	14	microT-CDS
Mucin type O-Glycan biosynthesis (hsa00512)	1.63E-03	13	10	microT-CDS

Supplementary data

S7 Table cont. Biological pathways enriched by differentially expressed microRNAs in Exo_D21 vs Exo_D3

KEGG pathway	p-value, FDR corrected	genes	miRNAs	algorithms
Long-term depression (hsa04730)	1.65E-03	32	13	microT-CDS
ErbB signaling pathway (hsa04012)	1.65E-03	44	14	microT-CDS
Glutamatergic synapse (hsa04724)	2.75E-03	50	13	microT-CDS
Glycosaminoglycan biosynthesis - heparan sulfate / heparin (hsa00534)	3.60E-03	12	8	microT-CDS
T cell receptor signaling pathway (hsa04660)	4.06E-03	49	14	microT-CDS
Platelet activation (hsa04611)	4.25E-03	55	15	microT-CDS
Regulation of actin cytoskeleton (hsa04810)	5.08E-03	93	17	microT-CDS
Endometrial cancer (hsa05213)	7.10E-03	28	12	microT-CDS
Long-term potentiation (hsa04720)	1.00E-02	33	13	microT-CDS
Acute myeloid leukemia (hsa05221)	1.36E-02	28	12	microT-CDS
Dorso-ventral axis formation (hsa04320)	1.46E-02	16	10	microT-CDS
Sphingolipid signaling pathway (hsa04071)	2.08E-02	51	13	microT-CDS
Dopaminergic synapse (hsa04728)	2.22E-02	57	14	microT-CDS
Fc epsilon RI signaling pathway (hsa04664)	2.47E-02	34	13	microT-CDS
Endocrine and other factor-regulated calcium reabsorption (hsa04961)	2.55E-02	23	12	microT-CDS
Fc gamma R-mediated phagocytosis (hsa04666)	2.74E-02	41	13	microT-CDS
Insulin signaling pathway (hsa04910)	2.83E-02	59	15	microT-CDS
Amphetamine addiction (hsa05031)	2.89E-02	29	12	microT-CDS
p53 signaling pathway (hsa04115)	2.92E-02	32	12	microT-CDS
Protein digestion and absorption (hsa04974)	2.92E-02	40	12	microT-CDS
cGMP-PKG signaling pathway (hsa04022)	2.92E-02	68	16	microT-CDS
Alanine, aspartate and glutamate metabolism (hsa00250)	2.96E-02	17	10	microT-CDS
Oxytocin signaling pathway (hsa04921)	3.17E-02	65	15	microT-CDS
Thyroid cancer (hsa05216)	3.34E-02	13	7	microT-CDS
Aldosterone-regulated sodium reabsorption (hsa04960)	3.34E-02	20	14	microT-CDS
Gap junction (hsa04540)	3.52E-02	36	13	microT-CDS
Viral carcinogenesis (hsa05203)	4.39E-02	67	14	microT-CDS
MicroRNAs in cancer (rno05206)	1.38E-90	132	15	Tarbase
Proteoglycans in cancer (rno05205)	2.05E-23	139	14	Tarbase
Hepatitis B (rno05161)	3.17E-11	95	14	Tarbase
Prostate cancer (rno05215)	1.24E-10	71	14	Tarbase
Glioma (rno05214)	1.43E-10	49	14	Tarbase
Chronic myeloid leukemia (rno05220)	1.04E-09	61	13	Tarbase
Renal cell carcinoma (hsa05211)	1.34E-09	54	13	Tarbase
Protein processing in endoplasmic reticulum (hsa04141)	6.56E-08	110	15	Tarbase
Fatty acid metabolism (hsa01212)	7.86E-08	30	13	Tarbase

Supplementary data

S7 Table cont. Biological pathways enriched by differentially expressed microRNAs in Exo_D21 vs Exo_D3

KEGG pathway	p-value, FDR corrected	genes	miRNAs	algorithms
Cell cycle (rno04110)	7.86E-08	86	14	Tarbase
Hippo signaling pathway (rno04390)	7.86E-08	88	14	Tarbase
ECM-receptor interaction (mmu04512)	8.26E-08	47	15	Tarbase
Pancreatic cancer (rno05212)	9.15E-08	50	13	Tarbase
Pathways in cancer (rno05200)	9.15E-08	231	15	Tarbase
TGF-beta signaling pathway (hsa04350)	1.05E-07	53	14	Tarbase
Bacterial invasion of epithelial cells (hsa05100)	1.36E-07	55	14	Tarbase
Non-small cell lung cancer (rno05223)	4.38E-07	41	13	Tarbase
Bladder cancer (rno05219)	5.46E-07	32	13	Tarbase
Small cell lung cancer (rno05222)	5.46E-07	62	15	Tarbase
FoxO signaling pathway (rno04068)	5.86E-07	92	14	Tarbase
Adherens junction (hsa04520)	7.00E-07	52	14	Tarbase
Lysine degradation (hsa00310)	1.04E-06	33	12	Tarbase
p53 signaling pathway (rno04115)	1.33E-06	53	14	Tarbase
Viral carcinogenesis (rno05203)	2.01E-06	123	14	Tarbase
Endometrial cancer (rno05213)	2.24E-06	40	14	Tarbase
Prion diseases (hsa05020)	3.33E-06	17	12	Tarbase
Focal adhesion (rno04510)	3.94E-06	130	15	Tarbase
Thyroid hormone signaling pathway (rno04919)	5.09E-06	75	14	Tarbase
Colorectal cancer (rno05210)	5.80E-06	47	14	Tarbase
TNF signaling pathway (hsa04668)	1.08E-05	73	15	Tarbase
Melanoma (rno05218)	1.44E-05	48	14	Tarbase
Central carbon metabolism in cancer (rno05230)	2.07E-05	47	14	Tarbase
Glycosaminoglycan biosynthesis - keratan sulfate (hsa00533)	2.11E-05	11	8	Tarbase
Other types of O-glycan biosynthesis (hsa00514)	2.11E-05	17	11	Tarbase
Fatty acid degradation (hsa00071)	3.29E-05	25	13	Tarbase
Signaling pathways regulating pluripotency of stem cells (hsa04550)	3.29E-05	86	15	Tarbase
Neurotrophin signaling pathway (hsa04722)	5.31E-05	76	14	Tarbase
Insulin signaling pathway (hsa04910)	5.32E-05	89	15	Tarbase
ErbB signaling pathway (hsa04012)	5.90E-05	57	15	Tarbase
Ubiquitin mediated proteolysis (hsa04120)	1.20E-04	83	15	Tarbase
Endocytosis (hsa04144)	1.48E-04	121	15	Tarbase
Shigellosis (hsa05131)	2.01E-04	42	13	Tarbase
PI3K-Akt signaling pathway (rno04151)	3.54E-04	178	15	Tarbase
Fatty acid biosynthesis (hsa00061)	3.55E-04	7	10	Tarbase
Prolactin signaling pathway (rno04917)	4.71E-04	46	13	Tarbase

Supplementary data

S7 Table cont. Biological pathways enriched by differentially expressed microRNAs in Exo_D21 vs Exo_D3

KEGG pathway	p-value, FDR corrected	genes	miRNAs	algorithms
Estrogen signaling pathway (hsa04915)	4.71E-04	61	14	Tarbase
Oocyte meiosis (hsa04114)	4.71E-04	63	15	Tarbase
Acute myeloid leukemia (rno05221)	7.03E-04	40	13	Tarbase
Thyroid cancer (rno05216)	8.48E-04	21	13	Tarbase
RNA transport (hsa03013)	1.44E-03	97	15	Tarbase
Epstein-Barr virus infection (hsa05169)	1.44E-03	118	15	Tarbase
Fatty acid elongation (hsa00062)	1.90E-03	13	8	Tarbase
Chagas disease (American trypanosomiasis) (hsa05142)	2.38E-03	61	14	Tarbase
Circadian rhythm (hsa04710)	2.66E-03	22	14	Tarbase
HIF-1 signaling pathway (hsa04066)	2.96E-03	66	15	Tarbase
Glycosaminoglycan biosynthesis - heparan sulfate / heparin (hsa00534)	4.20E-03	14	9	Tarbase
MAPK signaling pathway (hsa04010)	6.21E-03	137	14	Tarbase
Regulation of actin cytoskeleton (hsa04810)	6.36E-03	116	15	Tarbase
Hepatitis C (hsa05160)	8.87E-03	74	13	Tarbase
AMPK signaling pathway (rno04152)	8.95E-03	75	15	Tarbase
Apoptosis (hsa04210)	9.20E-03	52	14	Tarbase
Fc gamma R-mediated phagocytosis (hsa04666)	1.05E-02	54	14	Tarbase
Steroid biosynthesis (hsa00100)	1.11E-02	12	11	Tarbase
Axon guidance (hsa04360)	1.14E-02	68	15	Tarbase
mTOR signaling pathway (rno04150)	1.29E-02	40	15	Tarbase
Sphingolipid signaling pathway (rno04071)	1.38E-02	66	15	Tarbase
Progesterone-mediated oocyte maturation (hsa04914)	1.80E-02	52	14	Tarbase
Arrhythmogenic right ventricular cardiomyopathy (ARVC) (hsa05412)	2.01E-02	36	13	Tarbase
Transcriptional misregulation in cancer (hsa05202)	2.22E-02	98	14	Tarbase
Vitamin B6 metabolism (hsa00750)	2.27E-02	5	5	Tarbase
Dorso-ventral axis formation (hsa04320)	2.34E-02	19	15	Tarbase
Pathogenic Escherichia coli infection (hsa05130)	2.83E-02	34	15	Tarbase
Salmonella infection (hsa05132)	2.98E-02	49	15	Tarbase
Herpes simplex infection (hsa05168)	3.94E-02	104	14	Tarbase
Spliceosome (hsa03040)	4.72E-02	71	14	Tarbase
Measles (rno05162)	4.78E-02	75	14	Tarbase
Toxoplasmosis (hsa05145)	4.87E-02	65	15	Tarbase
